# Enhancing lettuce yield via Cu/Fe-layered double hydroxide nanoparticles spraying

**DOI:** 10.1186/s12951-023-02178-6

**Published:** 2023-11-11

**Authors:** Hongyang Wu, Xiaoyang Wan, Jiefei Niu, Huimin Xu, Yu Zhang, Xian Xue, Yang Li, Qiang Li, Tao Lu, Hongjun Yu, Weijie Jiang

**Affiliations:** 1 State Key Laboratory of Vegetable Biobreeding, Institute of Vegetables and Flowers, Chinese Academy of Agricultural Sciences, Beijing, 100081 China; 2 College of Horticulture, Xinjiang Agricultural University, Urumqi, 830052 China; 3 Research Unit of Molecular Epidemiology, Helmholtz Zentrum München, 85764 Neuherberg, Germany; 4 College of Biological Sciences, China Agricultural University, Beijing, 100193 China; 5 Faculty of Environmental Science and Engineering, Kunming University of Science and Technology, Kunming, 650500 China; 6 College of Agriculture, Henan University of Science and Technology, Luoyang, 471000 China

**Keywords:** Layered double hydroxides, Lettuce, Phenotypic analysis

## Abstract

Layered double hydroxides (LDHs) have been widely used in the field of plant engineering, such as DNA/RNA transformation and enhancing plant disease resistance. However, few studies have examined the direct effects of LDHs on plants and their potential utility as nanofertilizers. In this study, the retention capacity of Cu/Fe-layered double hydroxide nanoparticles (CuFe-LDHs) was assessed by comparative experiments on vegetables. The results showed that the retention of CuFe-LDHs in leafy vegetables was high, such as lettuce. Phenotypic analysis revealed that the fresh and dry weights of lettuce leaves were both increased by spraying 10–100 μg/mL CuFe-LDHs. Using the optimal concentration of 10 μg/mL, we conducted further experiments to elucidate the mechanism of CuFe-LDHs promoting lettuce growth. It was found that the application of CuFe-LDHs had a significant effect on growth and induced physiological, transcriptomic, and metabolomic changes, including an increase in the chlorophyll *b* content, net photosynthetic rate, and intercellular carbon dioxide concentration, as well as modifications in gene expression patterns and metabolite profiles. This work provides compelling evidence that CuFe-LDHs can efficiently adsorb on the surface of lettuce leaves through hydrogen bonding, promote lettuce growth, mitigate the toxicity of heavy metal ions compared to their raw materials at the same concentration and offer a molecular-scale insight into the response of leafy vegetables to CuFe-LDHs.

**Graphical Abstract:**

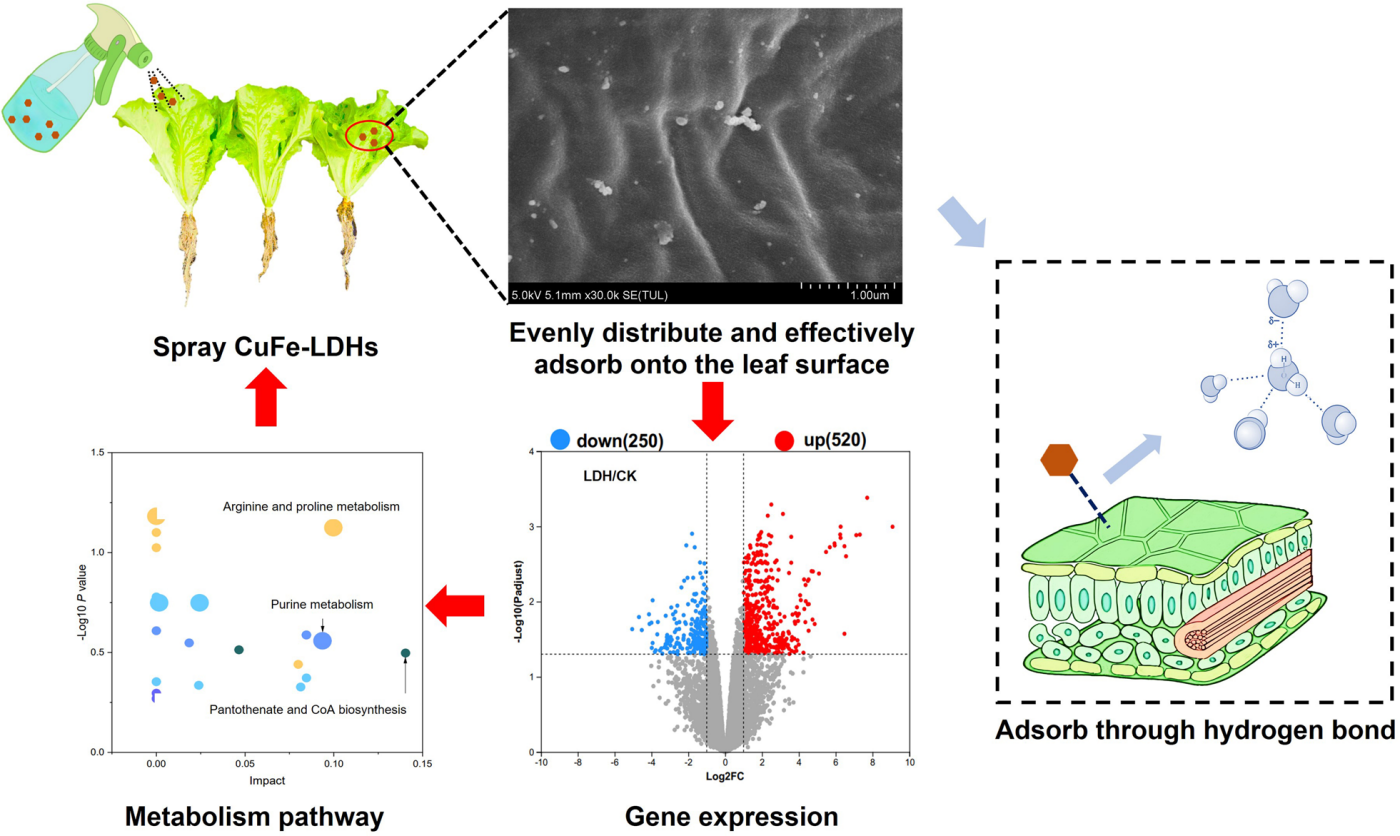

**Supplementary Information:**

The online version contains supplementary material available at 10.1186/s12951-023-02178-6.

## Introduction

Chemical and mineral fertilizers have ushered in a new era of agriculture, solved the historical challenge of food shortages, and substantially boosted global food production for centuries [1, 2]. However, the current cropping systems are facing significant challenges as a result of climate change and rapid population growth, which casted doubts on their long-term sustainability [3]. Contemporary agricultural practices rely heavily on traditional fertilizers for nutrients, but prolonged use of these fertilizers poses significant threats to the environment, soil fertility, and the nutritional balance of the rhizospheric microbiome [4, 5]. This can be attributed to the limited ability of plants to efficiently assimilate nutrients from conventional fertilizers or the inadequate and unbalanced nutrient composition of these fertilizers [6, 7]. Agricultural production urgently needs to utilize new technologies and develop more new materials to reduce the amount of organic and inorganic compounds, and improve crop yields and increase stress tolerance [2, 5, 7].

Nanomaterials have become unique tools for agricultural technology research due to their exceptional physicochemical properties [2, 8, 9]. Compared to conventional fertilizers, nanomaterials exhibit the capability to regulate nutrient release rates, enhance plant nutrition, improve crop absorption efficiency and diminish the unfavorable effects stemming from traditional fertilizer use (such as volatilization and eutrophication), thereby conferring high practical value [1]. The study of the biological effects of nanomaterials on plants originated from scientists' concerns about the potential negative impacts that caused by the extensive use of nanomaterials [10, 11]. To evaluate the influence of nanomaterials on plants and ecology, nanotoxicology researches have been conducted. Researchers found that nanomaterials can modulate plant growth, development, and metabolism, but their effects depend on factors such as category, physicochemical properties, concentration, and crop treatment conditions, leading to positive or negative outcomes [6, 10–13]. For instance, incorporating ZnO nanoparticles (NPs) into the peat moss:perlite (1:1) substrate led to noteworthy enhancements in both biomass production and the concentrations of chlorophyll and antioxidant compounds including phenolics, flavonoids, and vitamin C in lettuce [14]. Meanwhile, studies had shown that incorporating 5–250 mg/L ZnO nanoparticles (NPs) into hydroponic nutrient solution suppressed lettuce growth, reduced biomass, and hinders photosynthesis by affecting mineral nutrient absorption, chlorophyll synthesis, and photosystem II activity with increasing ZnO NPs concentration [15]. The above studies indicated that identical nanomaterials can have varying biological effects on a given vegetable depending on cultivation conditions. In summary, for optimal results with nanomaterials in plants, it’s crucial to identify and implement suitable cultivation conditions.

Among nanomaterials, layered double hydroxides (LDHs) possess distinctive features, such as facile synthesis, flexible structure regulation, excellent biocompatibility, low-cost, negligible cytotoxicity, facile biodegradation, and tunable release of encapsulated cargoes [16–18]. These traits qualify LDHs as promising candidates for various plant science applications, including nano pesticides, nano fertilizers, nanomedicine carriers, and plant nano gene carriers [19–21]. Wang et al. successfully synthesized and delaminated a solution of positively charged Mg/Al LDH nanosheets that are both stable and homogeneous in solution, exhibiting a thickness of 0.5–2 nm and a particle of 30–60 nm [21]. Subsequent studies have demonstrated that the aforementioned LDHs are highly effective as plant nanocarriers for various biomolecules, including ssDNA and fluorescein isothiocyanate (FITC) [20], long non-coding RNAs (lncRNAs) [22], circular RNAs (circRNAs) [23], and actinobacteria microorganisms [24], delivering them into plant cells. This is due to that larger LDHs can be adsorbed onto the cell wall for slower release of interlaminar anions, while smaller LDHs can penetrate the cell wall and enter plant cells via endocytosis or non-endocytic pathways [25, 26]. LDHs can also be used for plant disease resistance, with the first reported application in 2017: LDH-RNA bioconjugate was used to protect double-stranded RNA (dsRNA) from degradation when sprayed on tobacco leaves [27]. By spraying LDH nanosheets loaded with pDNAs expressing amiRNAs, Li et al. developed a method for efficiently preventing *tomato yellow leaf curl virus* (TYLCV) infection in tomato plants [28]. Mitter et al. demonstrated that using BioClay platform (combining dsRNA with MgFe-LDHs to form BioClay) to induce gene silencing through spray application effectively increased the mortality rate of cotton bollworms, achieving non-transgenic plant resistance against the pest [29]. Despite extensive research on the applications of LDHs, there has been little attention paid to their potential benefits, impact, and related mechanisms on plant growth and development.

In the present study, we synthesized and characterized Cu/Fe-layered double hydroxide nanoparticles (CuFe-LDHs) based on the following reasons: (i) Copper (Cu) and Iron (Fe) are known to play crucial roles in plant growth and development [30]; (ii) Cu and Fe deficiency are common issues observed in several economically important cash crops, including tea, barley, rose, and jasmine [31, 32]; (iii) Furthermore, Cu^2+^ ions possess fungicidal properties, making copper nanomaterials a promising candidate for integrating pesticides and fertilizers [33]. Subsequently, we investigated the effects of spraying CuFe-LDHs on lettuce growth and its related indexes. By phenotypic analysis, we determined the optimal concentration of CuFe-LDHs. Furthermore, we analyzed the ultrastructure of lettuce leaves treated with the optimal concentration of CuFe-LDHs by scanning electron microscopy (SEM) and transmission electron microscopy (TEM). For the first time, we elucidated the potential molecular mechanisms underlying the promotion of photosynthesis and yield in lettuce leaves by CuFe-LDHs through a comprehensive analysis of transcriptomic and metabolomic responses. The findings of this study significantly contribute to enhancing the understanding of the mechanism through which CuFe-LDHs promotes photosynthesis and yield. These results also provide a robust basis for the development of CuFe-LDHs nanofertilizers aimed at improving lettuce yields.

## Experimental section

### Preparation and characterization of CuFe-LDHs

The synthesis of CuFe-LDHs followed the procedure described by Laipan [34], with slight modifications of our own. To prepare CuFe-LDHs, a solution containing 100 mmol Cu(NO_3_)_2_ (99.99%; Macklin, China) and 50 mmol Fe(NO_3_)_3_ (99.99%; Macklin, China) in 100 mL deionized water (ddH_2_O) was sonicated and stirred. Additionally, a 100 mL solution of 4 mol/L NaOH (99%; Macklin, China) was prepared separately. Under nitrogen protection and with vigorous stirring at room temperature, both solutions were gradually added dropwise into a three-necked round-bottom flask containing 40 mL ddH_2_O, while maintaining the pH of the reaction mixture at 5.5 ± 0.2. The resulting viscous gel was then crystallized at 75 °C for 12 h in a completely sealed environment. Following crystallization, the product was filtered and washed until the supernatant reached a pH near 7. Finally, the solid was dried at 60 °C to obtain the final product CuFe-LDHs. The elemental contents of Cu and Fe in CuFe-LDHs were quantitatively analyzed by ICP-OES technology. The raw material (RW) consisted of Cu(NO_3_)_2_ and Fe(NO_3_)_3_ in quantities consistent with the amount of elemental Cu and Fe in the final product CuFe-LDHs. For X-ray diffraction (XRD) analysis, the heated products’ patterns were obtained using a Bruker D8 ADVANCE diffractometer equipped with Cu K*α* radiation, operating at 40 kV and 40 mA. The patterns were recorded over a 2*θ* range from 10° to 70°, with a scan speed of 5°/min. The size was measured by TEM, and the zeta potentials of CuFe-LDHs (1 g/L) were measured using a Malvern 2000 zeta potential analyzer (Malvern Instruments, UK).

### SEM images of CuFe-LDHs on leaves surfaces

To observe the surface structure of the leaves after spraying, a scanning electron microscope technique was employed. Lettuce leaves that had been growing for 2 weeks were selected and sprayed with H_2_O, 10 μg/mL CuFe-LDHs, or 10 μg/mL RW. After drying, samples were taken by using a double-sided blade and tweezers, with each sample measuring approximately 5*5 mm. These samples were then fixed in 2.5% (v/v) glutaraldehyde for 4 h. Next, the fixed samples were washed 3 times with 100 mM phosphate buffer saline (PBS; pH 7.2). Dehydration was carried out using a series of graded ethanol concentrations (30%, 50%, 70%, 85%, 95%, 100%). The samples were subsequently transferred to tert-butanol (Sigma, USA) and dried on copper wire at 4 °C. Finally, the samples were observed under a SU8020 scanning electron microscope (SEM; Hitachi, Japan).

#### Retention rate and adsorption mechanism of CuFe-LDHs on lettuce leaves surfaces

The leaf adhesion characteristics of CuFe-LDHs materials were evaluated using an indirect measurement method. The specific procedure involved dissolving centrifuged and purified CuFe-LDHs in a 1 g/L solution, and determining the concentration of Cu element (C1) using ICP-OES. Subsequently, 1 mL of H_2_O, CuFe-LDHs or RW was uniformly sprayed onto lettuce leaves (*Lactuca sativa*; Italy Elite Lettuce; Huiren, China) using a spray gun after ultrasonic dispersion. The droplets were left to dry at room temperature. To simulate the washing effect of rainwater on the surface of lettuce leaves, 100 mL ddH_2_O was vertically flushed onto the lettuce leaves. The resulting solution volume (V) was collected, and the concentration of Cu element (C2) was measured using ICP-OES. The amount of LDHs material retained on the crop leaves was calculated with three repetitions for each crop using the retention rate formula: Rr = 1-C1/(V*C2). To investigate the mechanism of CuFe-LDHs adsorption on the leaf surface, the aforementioned steps were repeated, and the crop leaves were subjected to vertical flushing with 100 mL of ddH_2_O, as well as 1%, 5%, and 10% urea solutions. To compare the retention rate of CuFe-LDHs on various vegetables, the above steps were repeated by evenly applying a uniform spray of 1 mL CuFe-LDHs on fruit vegetables such as tomatoes and cucumbers, as well as leaf vegetables like rapeseed and lettuce.

#### Plant material, growth conditions and content of elements in the leaves of lettuce in different treatments one month after the initial spray

The experiment was conducted at the China Academy of Agricultural Sciences in Beijing, China (116.33°E, 39.96°N). *Lactuca sativa* seeds were sterilized using a solution of 68% EtOH and 20% H_2_O_2_ (30%) for 5 min. The sterilized seeds were then planted on a nursery substrate consisting of turf, vermiculite, and perlite. The seedlings were covered with black plastic bags to block out light for 3 days and subsequently allowed to grow under normal conditions for 4 days. Afterwards, seedlings with similar growth status were carefully selected and transplanted into a coir substrate. At the same time, lettuce plants were subjected to spray treatments without or with 1, 10, and 100 μg/mL CuFe-LDHs, as well as 1, 10, and 100 μg/mL RW. Each treatment group consisted of ten lettuce plants, and a volume of 100 mL of solution was sprayed twice a week. The culture solution was poured twice a week, and the nutrient solution formula is provided in Additional file 1: Table S1.

One month after the initial application of CuFe-LDHs and RW, various parameters were measured to assess the growth and yield of lettuce. These parameters included fresh weight and dry weight of leaves, moisture content, leaves number, plant height, and plant width. Before separating the roots and leaves of each lettuce plant, leaves number, plant height, and plant width were recorded. The leaves were then rinsed with ddH_2_O and weighed to determine their fresh weight. After drying for a week at 60 °C, the leaves were weighed again to calculate their dry weight and calculate their moisture content.

The elemental content of control check (CK), 10 μg/mL CuFe-LDHs (LDH10 for short), and 10 μg/mL RW (RW10 for short) was measured using ICP-OES. From each treatment group, six vigorously growing plants were randomly selected, and the leaf with the largest surface area was chosen as the experimental material for each plant. These selected leaves were collected, mixed, dried, and ground before sampling. Each measurement was repeated 3 times. The concentrations of the following elements in lettuce were determined using ICP-OES: Ag, Ho, Yb, Pd, Sc, Tb, Be, Lu, Tm, Rh, Bi, Tl, Ga, Ir, Se, W, Pr, Sm, V, Y, Dy, Nd, Ni, Zr, Ge, Nb, Hg, Gd, La, Ta, Cd, Co, Eu, Pb, Er, As, Pt, Te, Mo, Au, Sb, Li, In, Ru, Ce, Hf, Cr, Ti, Cu, Ba, Sr, Sn, B, Si, Mn, Zn, Al, Fe, S, Mg, P, Ca, Na, K. Only elements with concentrations exceeding 1 mg/kg were selected for statistical analysis.

#### Photosynthetic characteristics, antioxidant system, and ultrastructure of lettuce leaves two weeks after initial spray

Two weeks after the initial application, the lettuce plants treated with CK, LDH10, and RW10 were utilized as experimental materials to evaluate their photosynthetic characteristics, antioxidant system, and ultrastructure. For the photosynthetic characteristics experiment, a portable photosynthesis measurement system Li-6400 (LICOR, USA) was employed. Real-time measurements were conducted using an artificial light source with a flux density of 800 μmol CO_2_ m^−2^ S^−1^. The flow rate was set at 1200 μmol S^−1^, while the leaf chamber temperature was maintained at room temperature. The measurement area was standardized to 6 cm^2^, targeting the leaf with the largest exposed surface area per lettuce plant. The parameters measured included net photosynthetic rate (Pn), stomatal conductance (GS), intercellular CO_2_ concentration (Ci), and transpiration rate (Tr). To ensure technical replication, each treatment group consisted of five sampled plants, with each plant being measured 5 times.

To assess antioxidant activity, leaf samples (0.10 g per sample) were immediately flash-frozen in liquid nitrogen and homogenized into a fine powder. The powdered samples were then transferred to a centrifuge tube containing 2 mL of extraction buffer and centrifuged at 8000 g for 10 min at 4 °C. The resulting supernatant was collected and kept on ice for further measurements. The activities of superoxide dismutase (SOD), peroxidase (POD), and catalase (CAT) were determined using assay kits obtained from Beijing Solarbio Science & Technology Co., Ltd. (Solarbio, China), following the provided instructions. To ensure representative sampling, each treatment group included four plants, with the careful selection of the leaf possessing the largest surface area from each plant.

To examine the leaf structure, ultra-thin sectioning and electron microscopy techniques were employed. Leaves measuring approximately 1*2*4 mm per sample were carefully sampled using a double-sided blade and tweezers, followed by three washes with 10 mmol/L EDTA-2Na. The samples were then fixed in 2.5% (v/v) glutaraldehyde for 4 h. After fixation, they were rinsed with 100 mM phosphate buffer saline (PBS; pH 7.2) and post-fixed in 1% osmium tetroxide for 2 h. Dehydration was carried out using a series of ethanol concentrations (30%, 50%, 60%, 70%, 80%, 90%, 95%, and 100%), followed by transfer to acetone. Finally, the samples were embedded in Spurr's resin (Sigma, USA). Sections from a minimum of ten leaves per treatment were cut using an LKB-V ultramicrotome, stained with 2% uranyl acetate (w/v) in 70% methanol (v/v) and 0.5% lead citrate. The samples were examined using a JEM-1230 transmission electron microscope (TEM; Tokyo, Japan) operating at 80 kV.

#### Transcriptome sequencing of lettuce leaves two weeks after initial spray

The experiment extracted RNA and characterized the transcriptome of lettuce leaves after the first LDH10 and RW10 spraying for 2 weeks. For each treatment group, six vigorously growing plants were randomly chosen, and the leaf with the largest surface area was selected as the experimental material from each plant. To ensure the removal of contaminants, the leaf surfaces were thoroughly rinsed with DEPC water, and any residual moisture was absorbed using filter paper. The leaves were then diced into small cubes, approximately 0.5 cm in size (similar to a soybean). After homogenization, the samples were divided into 100 mg aliquots and placed into RNase-free cryovials (Biosharp, China). These cryovials were rapidly frozen in liquid nitrogen for 0.5 h before being transferred to self-sealing bags for storage at − 80 °C in a freezer for preservation. The transcriptome sequencing experiment utilized the Illumina Novaseq 6000 sequencing platform (Illumina, USA) and followed the Illumina TruSeqTM RNA sample prep Kit method for library construction. To identify differentially expressed genes (DEGs), genes/transcripts with a false discovery rate below 0.05 and an absolute fold change ≥ 2 were considered significant. Further details of the experimental workflow, read mapping and the data analysis can be found in Additional file 1: Section S1. The raw sequence data of the samples were uploaded to NCBI at https://www.ncbi.nlm.nih.gov/sra/PRJNA1000780 (SUB13720407).

#### Metabolomics analysis of lettuce leaves two weeks after initial spray

The sampling method was consistent with the transcriptome sampling method. A 100 μL sample was transferred to a 1.5 mL EP tube, followed by the addition of 300 μL of methanol and 20 μL of internal standard. The mixture was vortexed for 30 s and subjected to 10 min of ultrasonication at 0 °C. Following this, it was cooled to − 20 °C. After this treatment, the samples were further cooled at − 20 °C for 1 h and centrifuged at 13,000 g for 15 min at 4 °C. Carefully, 200 μL of the supernatant was extracted and transferred into a 2 mL injection bottle. From each sample, 20 μL was taken and mixed to create a Quality Control (QC) sample. Subsequently, 200 μL of the mixture was carefully transferred into sample vials for ultraperformance liquid chromatography/tandem mass spectrometry (UPLC-MS/MS). The lettuce leaf extracts underwent metabolic analysis using UPLC-MS/MS to examine the effects of LDH10 and RW10. More information about the measurement and data analysis can be found in the Additional file 1: Section S2. The raw data for the samples have been uploaded to Metabolights (https://www.ebi.ac.uk/metabolights) with the accession number MTBLS8323.

### Real-time quantitative analysis

The sampling method was consistent with the transcriptome sampling method. Leaf tissue was harvested and flash-frozen in liquid nitrogen. Total RNA was extracted using the RN53 Total RNA extraction kit (Aidlab Biotechnologies, China) and reverse transcribed using the One-Step gDNA Removal and cDNA Synthesis SuperMix (TransGen Biotech, China). PCR was performed in an optical 96-well plate using the SYBR Green Mix (TransGen Biotech, China) and the ABI PRISM 7300 system (Bio-Rad, USA), following the manufacturer's instructions. Each reaction included 0.3 μM primer and 10 ng of cDNA. PCR amplification was carried out in triplicate for each of the four biological replicates. The PCR protocol consisted of an initial denaturation step of 5 min, followed by 40 cycles of denaturation at 95 °C for 20 s, annealing at 54 °C for 20 s, and extension at 72 °C for 20 s. A final extension step was performed at 84 °C for 30 s, followed by elongation at 72 °C for 10 min. The PCR cycles were followed by a melting curve analysis. Four independent biological replicates were analyzed using the comparative Ct method, as described in the iCycler manual (Bio-Rad, USA), to determine the relative mRNA expression levels in each tissue. Actin served as the internal control due to its similar amplification efficiencies compared to the target genes being analyzed (LOC111908221, LOC111899016, LOC111886489, LOC111899011, LOC111899010). The specific primers used in qRT-PCR are provided in Additional file 1: Table S2.

### Statistical analyses

The data were statistically processed using analysis of variance (ANOVA). Significant differences among the mean values were determined at a significance level of *p* < 0.05 by applying the least significant difference (LSD) test using SPSS and Excel software. The figures were created using Prism 9.0 software.

## Results and discussion

### XRD results of CuFe-LDHs and its retention rate on the leaf surface

**Fig. 1:**
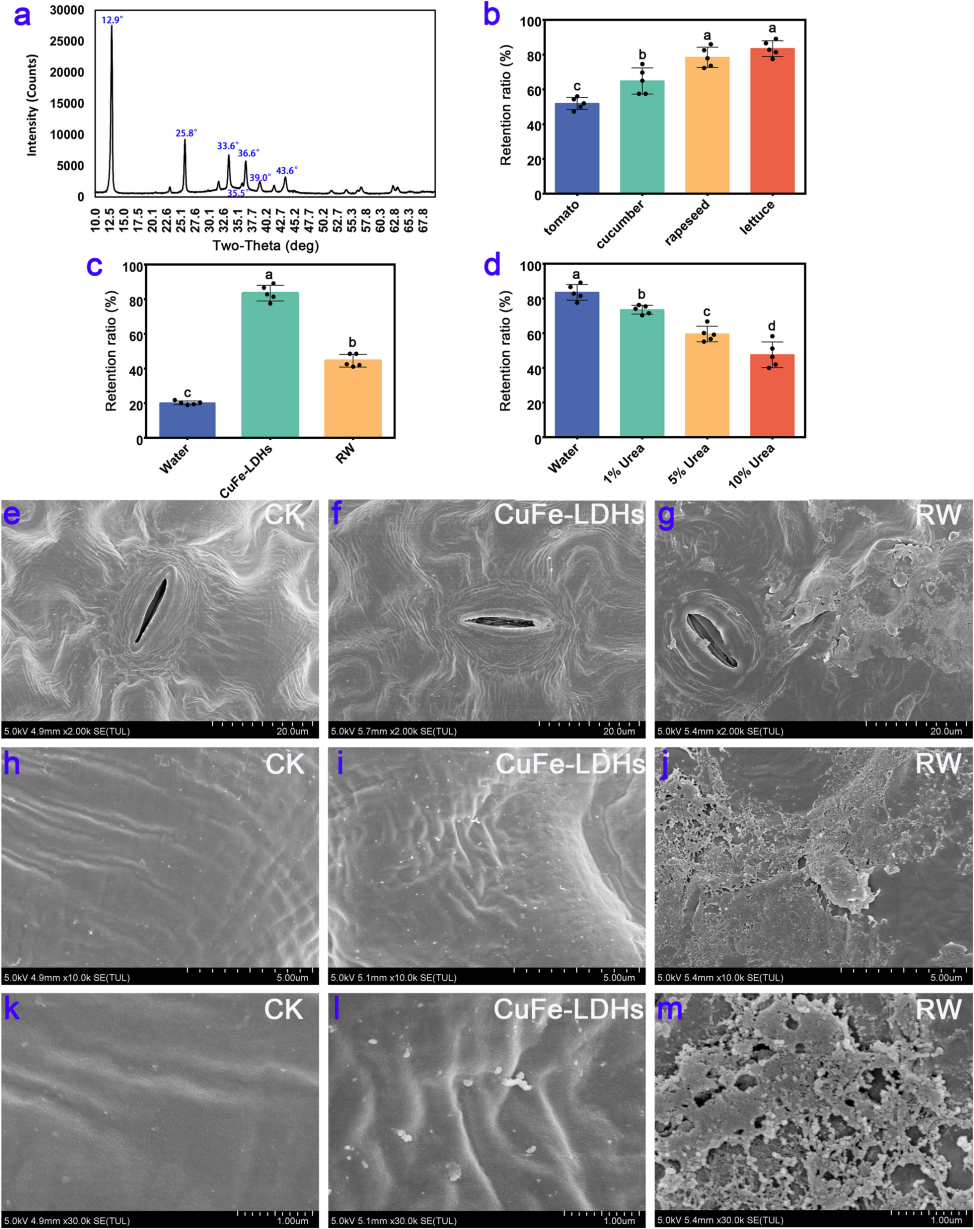
XRD results of CuFe-LDHs and their retention rate on the leaf surface. **a** XRD results of CuFe-LDHs. **b** The retention ratio on different plant leaves of 1 g/L CuFe-LDHs. **c** The retention ratio on the lettuce leaves of ddH2O, 1 g/L CuFe-LDHs, and 1 g/L RW. **d** The retention ratio of 1 g/L CuFe-LDHs on lettuce in different treatments. **e**–**m** SEM results of lettuce. CK, Control check. **e**, **h**, **k** CK, **f**, **i**, **l** 10 μg/mL CuFe-LDHs, **g**, **j**, **m** 10 μg/mL RW

We conducted an XRD analysis on the synthesized CuFe-LDHs, and the results are shown in Fig. 1a. When the molar ratio of copper (Cu) to iron (Fe) was maintained at 2:1, distinct diffraction peaks corresponding to specific crystal planes of the layered structure, namely (003), (006), and (009), were observed at angles of 2*θ* = 12.9°, 25.8°, and 33.6°, respectively. These peaks exhibited well-defined profiles, indicating a high level of crystallinity. Additionally, the presence of the LDH structure was confirmed by the detection of diffraction peaks at 2*θ* = 36.6° and 43.6°, which can be attributed to the crystal planes of (015) and (018) [35]. Compared with the conventional LDH structure, the diffraction peaks of CuFe-LDHs were shifted upward at higher angles, primarily due to the Jahn–Teller effect induced by divalent copper ions, which results in structural distortion [36]. Furthermore, additional diffraction peaks at 2*θ* = 35.5° and 39.0° were observed, corresponding to the standard card (JCPDS: 48–1548), and this suggested the presence of a low quantity of monoclinic copper oxide (CuO) impurities in the synthesized material. Previous study showed that CuO NPs were more toxic than the Cu^2+^ [37]. Nevertheless, at low concentrations (< 2 mg/L), CuO NPs had no detrimental effect on plant growth [38]. Consequently, we can conclude that the presence of a low quantity of CuO impurities will not interfere the effects of CuFe-LDHs in plants. The TEM results, as shown in Additional file 1: Fig. S1, revealed that on the carbon film, CuFe-LDHs particle sizes range from approximately 30–100 nm. In order to confirm that the CuFe-LDHs dispersion are stable, zeta potential analyses are performed. The results, as shown in Additional file 1: Fig. S2, showed that the zeta potential of CuFe-LDHs dispersions was + 24.2 mV, suggesting that CuFe-LDHs are positive charged and the dispersions are relatively stable. As shown in Additional file 1: Fig. S3, the particle size of CuFe-LDHs was 60.1 nm. Both Zeta potential and particle size exhibit a normal distribution with a single peak, indicating that particles in the solution possess similar size and charge distributions, are uniformly dispersed in the solution, and exhibit high stability.

To investigate whether LDH adheres to the leaves, we used simulated rainwater flushing to detect the retention of CuFe-LDHs on the leaf surface before and after rainwater flushing. After vertical flushing with 100 mL of ddH_2_O, the retention rates of CuFe-LDHs on the tomato, cucumber, rapeseed, and lettuce leaf surfaces were 51.9 ± 3.47%, 64.86 ± 7.53%, 78.48 ± 5.83%, and 83.48 ± 4.52%, respectively (Fig. 1b). The above results indicate that CuFe-LDHs show high retention on lettuce leaf surfaces. There were significant differences between the retention rates of CuFe-LDHs on the above leaf surfaces, ranging from 51.9 ± 3.47% to 83.48 ± 4.52%. The reason for this difference in retention rates can be attributed to leaf surface structure and waxes on leaf surfaces, which exhibit diverse characteristics among different species, classified as either weakly hydrophobic or strongly hydrophobic traits [39]. Consequently, this leads to varying retention rates when applying different species of leaves during spraying the same liquid [39, 40]. Comparison of the foliar retention of CuFe-LDHs and their raw materials on lettuce leaves revealed that the foliar retention of H_2_O, CuFe-LDHs, and their raw materials (RW) was 20.16 ± 1.11%, 83.48 ± 4.52%, and 44.52 ± 3.67%, respectively, after being washed with 100 mL of ddH_2_O (Fig. 1c). The retention of CuFe-LDHs on the surface of lettuce leaves far exceeded that of RW and water. To investigate the leaf surface adherence mechanism of CuFe-LDHs, LDHs were evenly applied to the leaves of lettuce. The leaves were washed with 100 mL of H_2_O and 1%, 5%, and 10% urea solutions, and the retention rates were 83.48 ± 4.52%, 73.48 ± 2.52%, 59.5 ± 4.47%, and 47.5 ± 7.36%, respectively (Fig. 1d). After gradient urea treatment, the retention rate of CuFe-LDHs on the surface of lettuce leaves decreased.

The adhesion phenomenon in nature is achieved via two mechanisms. (i) Adhesion is attained through the relative sliding at the contact interface facilitated by Van der Waals forces (e.g., geckos and spiders employ bristles on their feet to adhere to the contact surface). (ii) Adhesion is further strengthened at the contact interface by the secretion of substances that form hydrogen bonds (e.g., creeper plants secrete L-rhamnose to facilitate adhesion to the contact surface) [41]. Urea can destroy the hydrogen bonds inside protein molecules to promote their degradation [42]. After urea treatment, the retention of CuFe-LDHs was significantly reduced (Fig. 1d), indicating that the adsorption of LDHs on the leaf surface was determined by hydrogen bonding. The functional groups that can generate hydrogen bonds include hydroxy (-OH), amino (-NH_3_), carboxyl (-COOH), and carbonyl (C = O) [43]. As described in the "Experimental", CuFe-LDHs were prepared using two types of nitrates and strongly alkaline NaOH, which introduced a large number of hydroxyl groups during the preparation process. The number of hydroxyl groups on the unit area has a certain relationship with adhesion [44], a large number of hydroxyl groups enriched on the surface of the material can mediate the adsorption of blades. The retention rate of RW is much lower than that of CuFe-LDHs. The most likely explanation is that RW is composed of copper nitrate and iron nitrate, which cannot bind to the leaf surface via hydrogen bonding. In short, CuFe-LDHs composed of Cu and Fe elements were compared with the synthetic raw material copper nitrate and iron nitrate, which enhance the adhesion of the leaf surface.

To observe the surface structure of the leaves after spraying, two-week-old lettuce leaves were sprayed with H_2_O, 10 μg/mL CuFe-LDHs, or 10 μg/mL RW; the SEM results are shown in Fig. 1e–m. It is widely known that most airborne dust particles carry a negative charge [45], which makes them prone to being adsorbed by positively charged particles. The retention of RW on the blade surface was lower compared with CuFe-LDHs (Fig. 1c), which might be responsible for the inability of most of the charged particles (Cu^2+^, Fe^3+^) in RW10 to bind to the blade surface. Instead, they might adsorb anions or airborne dust particles on the blade surface, forming aggregates (Fig. 1g, j, m). In contrast, LDHs formed electrically neutral particles and evenly dispersed on the surface of the blades (Fig. 1f, i, l). Therefore, by employing a comparatively lesser quantity of CuFe-LDHs, superior outcomes can be attained compared to those obtained with RW.

### CuFe-LDHs promoted the growth of lettuce and affected the accumulation of elements one month after the initial spray

**Fig. 2:**
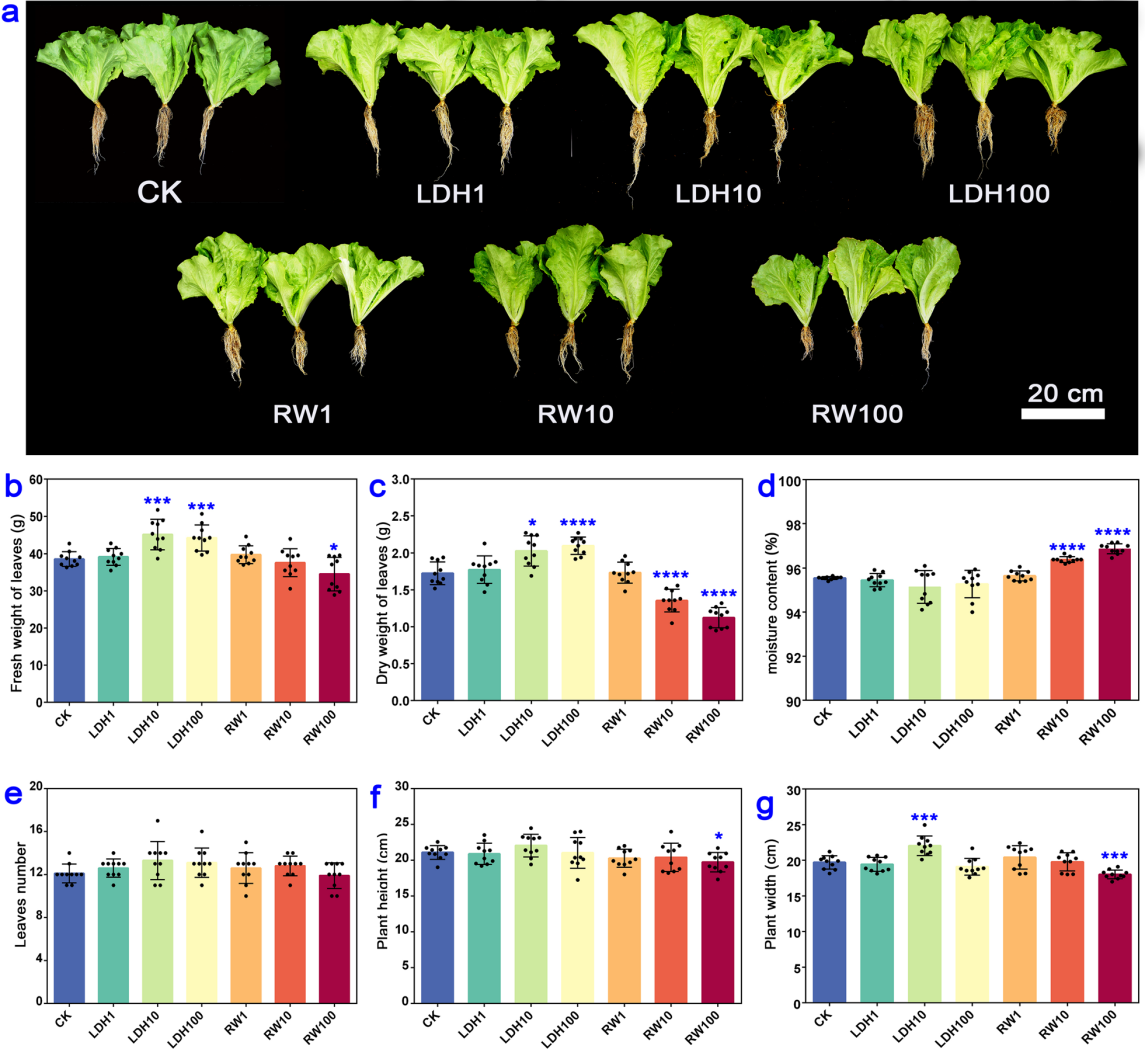
Effect of spraying different concentrations of CuFe-LDHs and RW on the phenotype of lettuce after one month. **a** Lettuce treated with varying concentrations of LDHs and RW. **b** Fresh weight of leaves. **c** Dry weight of leaves. **d** Moisture content. **e** Leaves number. **f** Plant height. **g** Plant width. Error bars represent SD. CuFe-LDHs = 1, 10, and 100 μg/mL correspond to LDH1, LDH10, and LDH100 for short. RW = 1, 10, and 100 μg/mL are RW1, RW10, and RW100 for short. *p < 0.05, ***p < 0.001, and ****p < 0.0001, Student’s *t*-test. Bar = 20 cm

To investigate the effect of spraying different concentrations of CuFe-LDHs on plant growth, we took measurements of various parameters, including the fresh and dry leaf weight, moisture content, leaves number, plant height, and plant width one month after the initial spray. We also established a control group using RW, which contained the raw materials of CuFe-LDHs at an equivalent concentration. The phenotypic characteristics of lettuce were presented in Fig. 2a. The application of CuFe-LDHs at concentrations of 10 μg/mL (45.15 ± 3.84 g) and 100 μg/mL (44.23 ± 3.30 g) significantly increased the fresh weight of lettuce (*p* < 0.001) compared with the CK group (38.59 ± 1.88 g) (Fig. 2b). Conversely, the application of RW at 100 μg/mL had a significant inhibitory effect on the fresh weight of lettuce (*p* < 0.05) (Fig. 2b). Similarly, the dry weight of lettuce was significantly higher in the 10 μg/mL (2.03 ± 0.19 g) (*p* < 0.05) and 100 μg/mL (2.10 ± 0.11 g) (*p* < 0.0001) CuFe-LDHs groups compared with the CK group (1.73 ± 0.15 g) (Fig. 2c). In contrast, both 10 µg/mL (1.36 ± 0.14 g) and 100 μg/mL (1.13 ± 0.13 g) RW inhibited increases in the dry weight (*p* < 0.0001) (Fig. 2c). Moreover, the moisture content of lettuce was significantly higher in the 10 μg/mL (96.38 ± 0.13%) and 100 μg/mL (96.84 ± 0.20%) RW groups than in the CK group (95.55 ± 0.06%) (*p* < 0.0001) (Fig. 2d). Leaves number was unaffected by the application of CuFe-LDHs or RW (*p* > 0.05) (Fig. 2e). Plant height was significantly lower in the 100 μg/mL RW (19.73 ± 1.28 cm) group than in the CK group (21.09 ± 0.91 cm) (*p* < 0.05) (Fig. 2f). Plant width (22.03 ± 0.30 cm) was significantly higher in the 10 μg/mL CuFe-LDHs group than in the CK group (19.69 ± 0.90 cm), and the application of 100 μg/mL RW (18.02 ± 0.58 cm) resulted in a significant reduction in plant width (*p* < 0.001) (Fig. 2g). Overall, these findings demonstrate that the application of CuFe-LDHs at concentrations of 10–100 µg/mL promotes growth, whereas RW application at the same range of concentrations hinders growth. One month after the initial spray, LDH10 increased the Cu content in lettuce within an appropriate range (Table 1). In light of the substantial increases observed in the fresh weight, dry weight, and leaf width achieved by the spraying of 10 μg/mL CuFe-LDHs compared with 100 μg/mL LDH, 10 μg/mL CuFe-LDHs was considered the optimal concentration.

**Table 1:** Content of elements in the shoots of lettuce under hydroponic culture in different treatments

	Large and medium element/(mg·kg^−1^)					
	K	P	Ca	Mg	S	Na
CK	62,128.9 ± 918.4c	13,189.5 ± 127.4a	14,369.4 ± 431.9a	4898.2 ± 415ab	2959.9 ± 90.2b	1331.9 ± 15.4 b
LDH10	76,322.7 ± 702.3b	10,372.8 ± 213.1b	13,388.5 ± 7.7ab	5581.7 ± 170.4a	2892.5 ± 90.1b	1913.1 ± 83.5 a
RW10	91,904.7 ± 1744.1a	10,390.2 ± 316.5b	11,276.1 ± 356.7b	4565.4 ± 41.3b	3531.9 ± 102.6a	2058.7 ± 22.8 a

	Trace element/(mg·kg^−1^)					
	Cu	Fe	Mn	Zn	Al	B
CK	5.5 ± 0.5b	103.6 ± 2.9b	69.1 ± 1.2a	60.2 ± 2.2b	55.1 ± 0.9a	18.8 ± 2.6b
LDH10	9.5 ± 0.5a	117.8 ± 3.3a	61.9 ± 1.6b	59.5 ± 2.2b	56.3 ± 1.2a	23.9 ± 1.1ab
RW10	11.0 ± 0.7a	118.9 ± 2.3a	52.7 ± 3.2b	82.8 ± 0.4a	39.1 ± 1.4b	28.9 ± 1.8a

Different lowercase letters in the same column indicate significant differences at *p* < 0. 05 between treatments. Three technical replicates were performed for each treatment

### CuFe-LDHs enhance photosynthesis without affecting the antioxidant system and ultrastructure of lettuce leaves two weeks after the initial spray

**Fig. 3:**
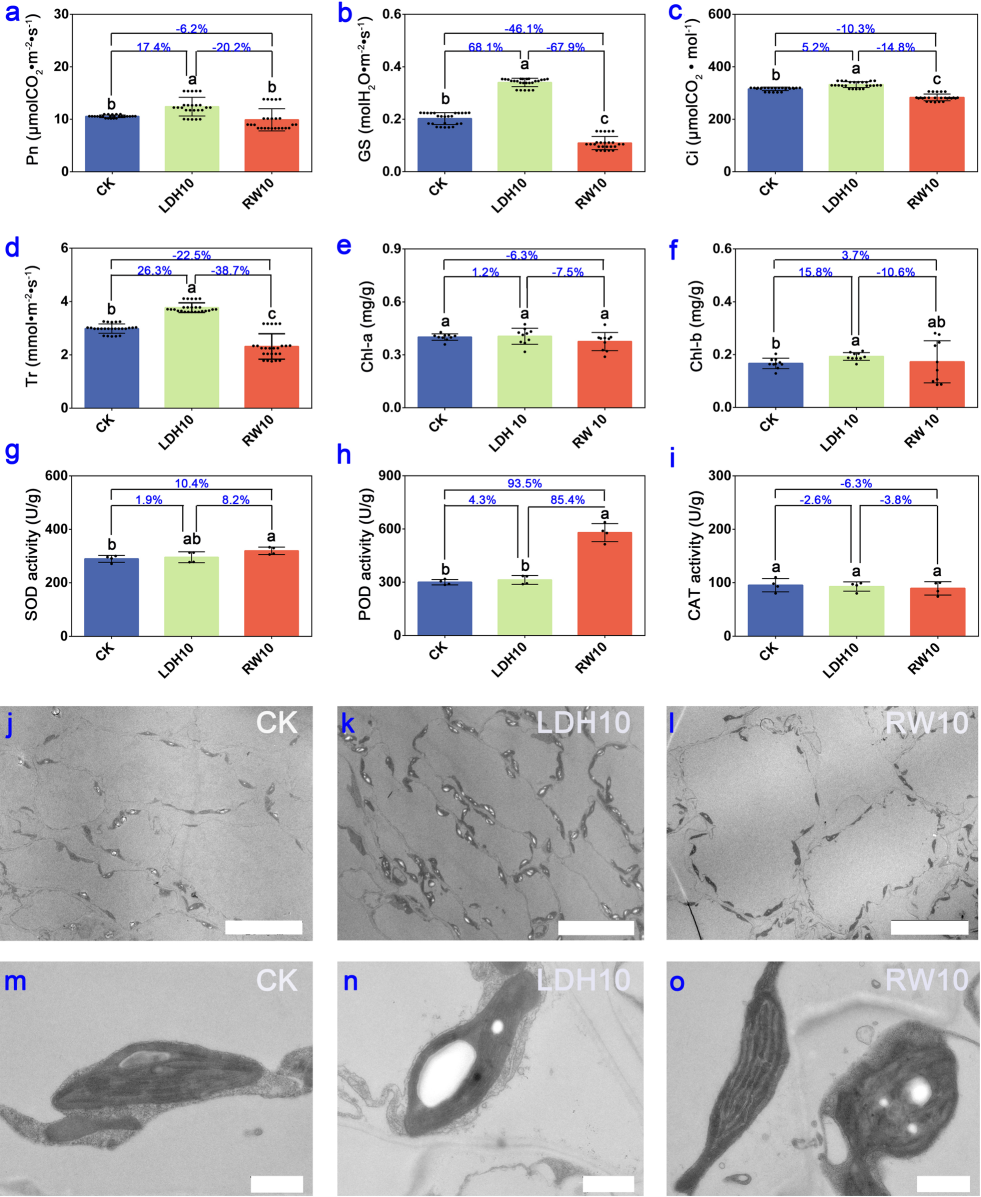
Photosynthetic characteristics, antioxidant system, and ultrastructure of lettuce leaves two weeks after the initial spray of LDH10 and RW10. Net photosynthetic rate (Pn, **a**), stomatal conductance (GS, **b**), intercellular CO_2_ concentration (Ci, **c**), and transpiration rate (Tr, **d**) of lettuce. The chlorophyll *a* content (chl-a, **e**), chlorophyll *b* content (chl-b, **f**), and superoxide dismutase (SOD, **g**), peroxidase (POD, **h**), and catalase (CAT, **i**) activity in lettuce leaves following foliar exposure to LDH10 and RW10. Different lowercase letters indicate significant differences among treatments (*p* < 0.05). The percentages show the magnitude of change among the different treatment groups (LDH10/CK, RW10/CK, LDH10/RW10). Bar = 20 μm **j**, **k**, **l**. Bar = 1 μm **m**, **n**, **o**. The abbreviations used are the same as in Fig. 2

To evaluate photosynthetic characteristics, antioxidant system, and ultrastructure, we utilized the lettuce plants treated with CK, LDH10, and RW10 two weeks after the initial application as experimental materials. Obviously, LDH10 and RW10 had a significant effect on photosynthesis after the initial spray for 2 weeks based on observed changes in multiple parameters, including net photosynthetic rate (Pn), stomatal conductance (GS), intercellular CO_2_ concentration (Ci), and transpiration rate (Tr) (*p* < 0.01) (Fig. 3a–d). These findings suggest that LDH10 significantly enhances photosynthesis in lettuce, whereas RW10 significantly inhibits photosynthesis. One of the factors contributing to this disparity is the uniform distribution of LDHs on the leaf surface, which allows them to bind to the leaves through hydrogen bonding (Fig. 1f, i, l). As a result, electrically neutral particles are formed that do not hinder stomatal function nor affect GS and Tr (Fig. 3b, d). Conversely, the physical shielding effect of RW10 hindered the photosynthetic reactions in lettuce leaves (Fig. 3a–d), which directly affected plant yield (Fig. 2b, c). Previous studies have highlighted the exceptional CO_2_ adsorption capability of LDHs, and this has made them extensively used in ongoing efforts to achieve carbon neutrality because of their high CO_2_ adsorption capacity [19]. In light of LDHs' capacity to enhance the Pn and intercellular CO_2_ absorption (Fig. 3a, c), we hypothesize that the main mechanism underlying the adsorption of CO_2_ by LDHs stems from its ability to increase the Ci. This increase in Ci results in the augmentation of the substrate, thereby promoting an increase in the Pn.

Furthermore, we speculate that the elevation of GS and Tr are related to the physiological activities of CuFe-LDHs after they enter plant cells. Previous studies have indicated that LDHs can overcome the barrier of the cell wall to enter plant cells [20, 25, 27, 29], and pass through the plasma membrane [25, 26]. Ultimately, LDHs undergoes decomposition in the elevated H^+^ environment within the cytoplasm or vacuole, slowly releasing the metal cations that make up the LDHs [46]. We can infer that CuFe-LDHs can enter cells and slowly degrade into low concentrations of Cu^2+^ and Fe^3+^ within cells, subsequently induce changes in the cellular physiological levels. Copper and iron are essential mineral elements for plant growth and development. Copper is a component of the photosynthetic electron transport chain, for CO_2_ assimilation and ATP synthesis [47], involved in photosynthetic reactions of PSII independent of plastocyanin [48]. Fe has greater importance in photosynthesis and respiration [49]. The photosynthetic machinery in plants is abundantly supplied with Fe atoms, comprising 12 Fe atoms per Photosystem I (PSI), 2 or 3 Fe atoms per Photosystem II (PSII), 5 Fe atoms within the cytochrome complex *b*_6_-f (cyt *b*_6_-f), and 2 iron atoms per ferredoxin molecule [50]. Therefore, photosynthetic organisms exhibit a high sensitivity to alterations in iron availability, leading to a significant reduction in photosynthetic activity when subjected to iron deficiency [51]. In summary, maintaining an appropriate concentration of Cu and Fe plays a pivotal role in enhancing the activities of PSI and PSII, facilitating CO_2_ assimilation, and promoting ATP synthesis. Moreover, within an optimal range, increasing iron content can elevate GS levels, thereby enhancing photosynthesis [52]. An increase in Fe content simultaneously enhances GS, which is consistent with our findings (Fig. 3b). In conclusion, we can infer that by gradually releasing low concentrations of Cu and Fe ions within cells, CuFe-LDHs influences the synthesis of proteins and enzymes related to photosynthesis, enhances GS and Pn, ultimately promoting photosynthesis.

No differences in the chlorophyll *a* content were observed among the LDH10, RW10, and CK treatments (Fig. 3e). LDH10 significantly increased the content of chlorophyll *b*; however, RW10 had no significant effect on the content of chlorophyll *b* (Fig. 3f). Chlorophyll* a* and its derivatives primarily absorb red light (620-700 nm), whereas chlorophyll *b* predominantly absorbs blue-violet light (400-500 nm) [53]. In light of the unique absorption spectrum of LDHs [54], we speculate that the presence of LDH10 on the leaf surface reduces the absorption of blue-violet light, thereby prompting the synthesis of chlorophyll *b* to enhance the absorption of blue-violet light.

Exposure to RW10 via foliage significantly increased the content of SOD (Fig. 3g) and POD (Fig. 3h) in lettuce leaves, which induced a stress response in the antioxidant system. However, there was no significant difference in the content of SOD (Fig. 3g), POD (Fig. 3h), or CAT (Fig. 3i) in lettuce in the LDH10 treatment, indicating that LDH10 does not induce stress responses in lettuce. The activity of antioxidant enzymes such as SOD, POD, and CAT plays a key role in scavenging excessive O^2−^ and H_2_O_2_, thereby mitigating damage caused by biotic or abiotic stress [55]. SOD activity was 10.4% higher in RW10 than in the CK (Fig. 3g), and POD activity was 93.5% higher in RW10 than in the CK (Fig. 3h). These results indicate that SOD and POD effectively eliminate accumulated H_2_O_2_ in lettuce leaves, reducing the levels of free radicals and alleviating membrane lipid peroxidation damage in older leaves. Similarly, hydroponically cultured lettuce produces a substantial amount of SOD within its tissues when subjected to external stress, which enables them to scavenge stress-induced superoxide radicals and protect the plants [56]. Furthermore, Cd stress significantly up-regulates POD enzyme genes in lettuce, which enhances resistance to Cd stress [57].

Ultrastructural analysis revealed that the number of transient starch granules was significantly higher in leaves in the LDH10 treatment (Fig. 3k) than in the CK (Fig. 3j), and the number of transient starch granules was significantly lower in leaves in the RW10 treatment (Fig. 3l) than in the CK (Fig. 3j), suggesting that the application of LDH10 and RW10 may affect photosynthesis. In the lettuce leaf cells of CK, chloroplasts appeared elliptical, thylakoids were arranged parallel to the long axis of the chloroplasts, grana stacks formed granal lamellae, and the stroma was uniform (Fig. 3m). The intact chloroplasts in LDH10 leaves (Fig. 3n) had well-organized thylakoids and clear granaf lamellae, similar to the normal chloroplasts in CK. In contrast, analysis of RW10-treated leaves revealed disorganized stacks of chloroplasts and the presence of malformed chloroplasts (Fig. 3o). These findings suggest that the LDH10 treatment does not affect the structure of chloroplasts in lettuce leaves. To our surprise, LDH10 was not detected using the ultra-thin sectioning method (Fig. 3k, n). Previous studies have indicated that LDHs can overcome the barrier of the cell wall to enter plant cells [20, 25, 27, 29]. LDHs enter plant cells through the following steps: (1) smaller-sized LDHs can penetration across cell wall. Larger-sized LDHs need to undergo delamination into smaller-sized LDHs or nanosheets in the presence of CO_2_ and humidity to pass through the cell wall barrier [20, 27]; (2) LDHs pass through the plasma membrane via non-endocytic pathways and endocytosis [25]; (3) LDH undergoes decomposition in the elevated H^+^ environment within the cytoplasm or vacuole [25]. The exact mechanism behind this phenomenon has yet to be elucidated. The most plausible explanation is that LDH10 adsorbed onto the cell wall and slowly degraded into the cells due to CO_2_ and humidity, as described in previous studies [27, 29]. Another possibility that cannot be ruled out is that smaller layers of LDHs delaminated and entered the cells [25], but the LDH particles could not be observed via TEM because of their small size. Studies of duckweed have demonstrated that low concentrations of Cu^2+^ can promote plant growth, whereas high concentrations of Cu^2+^ can inhibit plant growth by disrupting the structure of chloroplasts or thylakoids and reducing the activity of photosystem II [58]. Interestingly, we observed damage to the chloroplasts and thylakoids in RW10 (Fig. 3o), but not in LDH10 (Fig. 3n). The most plausible explanation was that LDH10 contains Cu^2+^ both in its free form and bound to LDH layers, and it releases Cu^2+^ slowly, thereby reducing its toxicity.

In light of the biomass, photosynthetic pigment, antioxidant enzyme activity, and intracellular structure of lettuce leaves, we conclude that physiological toxicity was higher and the stress response was stronger in RW10 compared with LDH10.

### Integrated transcriptome and metabolome analysis of lettuce leaves two weeks after the initial spray

**Fig. 4:**
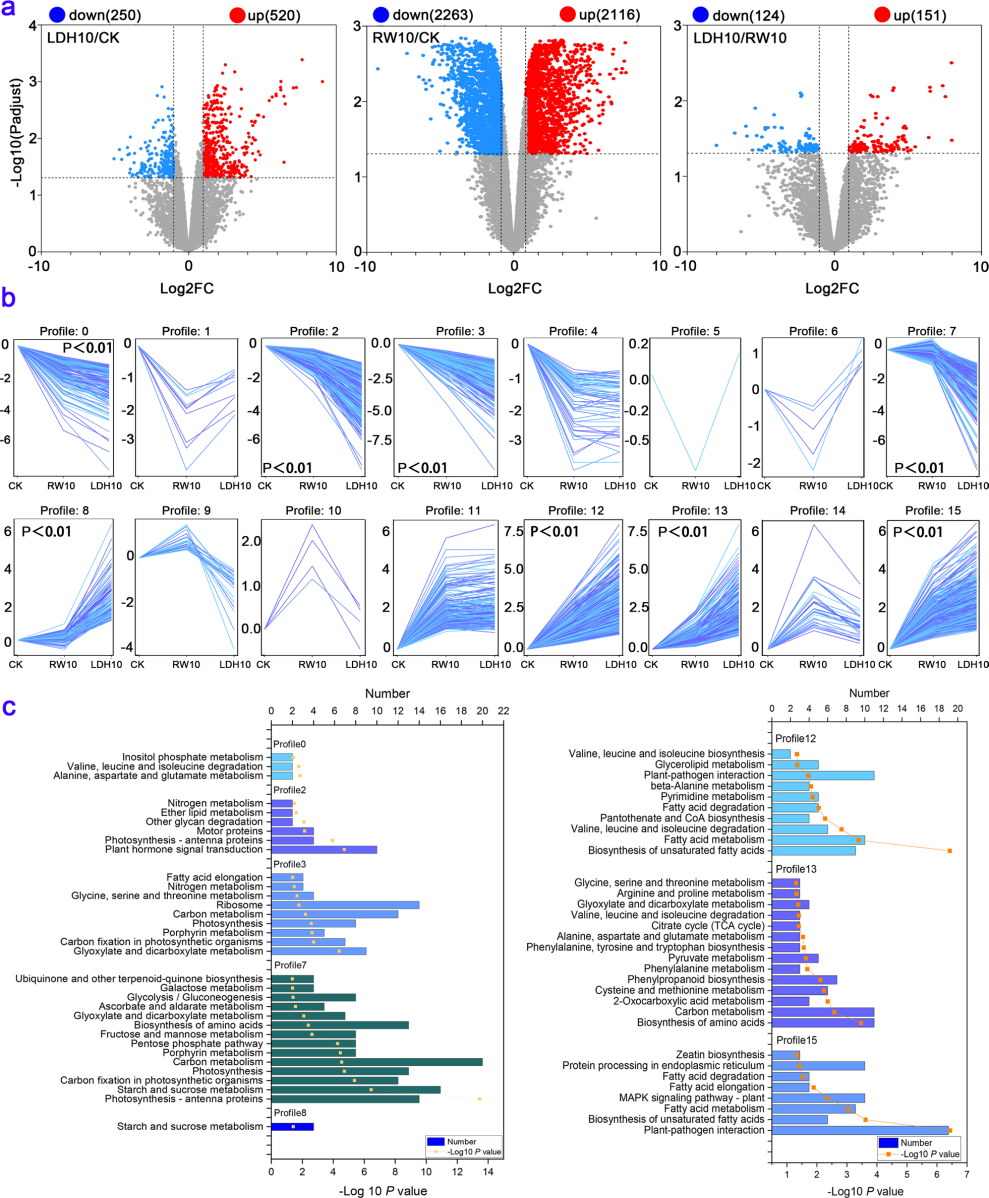
Transcriptome analysis of lettuce leaves after 2 weeks of initial treatment. **a** Volcano plots depicting the differentially expressed genes (DEGs) with a false discovery rate (FDR) below 0.05 and an absolute fold change of ≥ 2 between various treatment groups (LDH10/CK, RW10/CK, LDH10/RW10). **b** The expression patterns of DEGs across LDH10 and RW10 treatments were inferred using Short Time-series Expression Miner (STEM) analysis. Each frame represents the expression pattern of all the DEGs, which are indicated by the colored lines. **c** The Kyoto Encyclopedia of Genes and Genomes (KEGG) pathway analysis revealed significantly overrepresented profiles of differentially expressed genes in lettuce leaves in the LDH10 and RW10 treatments. The values are presented as the mean ± standard deviation of three replicates for each treatment. The abbreviations used are the same as in Fig. 2

To further investigate the potential molecular mechanism underlying the enhanced growth of lettuce after CuFe-LDH treatment, we conducted a transcriptome analysis of lettuce leaves that had been treated with or without CuFe-LDHs. A total of 770 DEGs (520 up-regulated and 250 down-regulated) were detected in leaves treated with LDH10 relative to the CK (Fig. 4a, Additional file 1: Fig. S4). Similarly, there were 4379 DEGs (2,116 upregulated and 2263 down-regulated) in leaves treated with RW10 relative to the CK (Fig. 4a). The number of up-regulated DEGs was higher than the number of down-regulated DEGs in the LDH10 treatment. Conversely, the number of up-regulated DEGs was lower than the number of down-regulated DEGs in the RW10 treatment (Fig. 4a). The total DEGs of LDH10 and RW10 were clustered into 16 profiles (from profile 0 to 15) based on gene expression patterns using Short Time-series Expression Miner software (Fig. 4b) to identify significantly changed DEGs. The most represented clusters were profiles 0, 2, 3, 7, 8, 12, 13, and 15 (*p* < 0.01). To gain further insights into transcriptional changes, KEGG enrichment analysis was performed for genes belonging to profiles 0, 2, 3, 7, 8, 12, 13, and 15 (Fig. 4c).

**Fig. 5:**
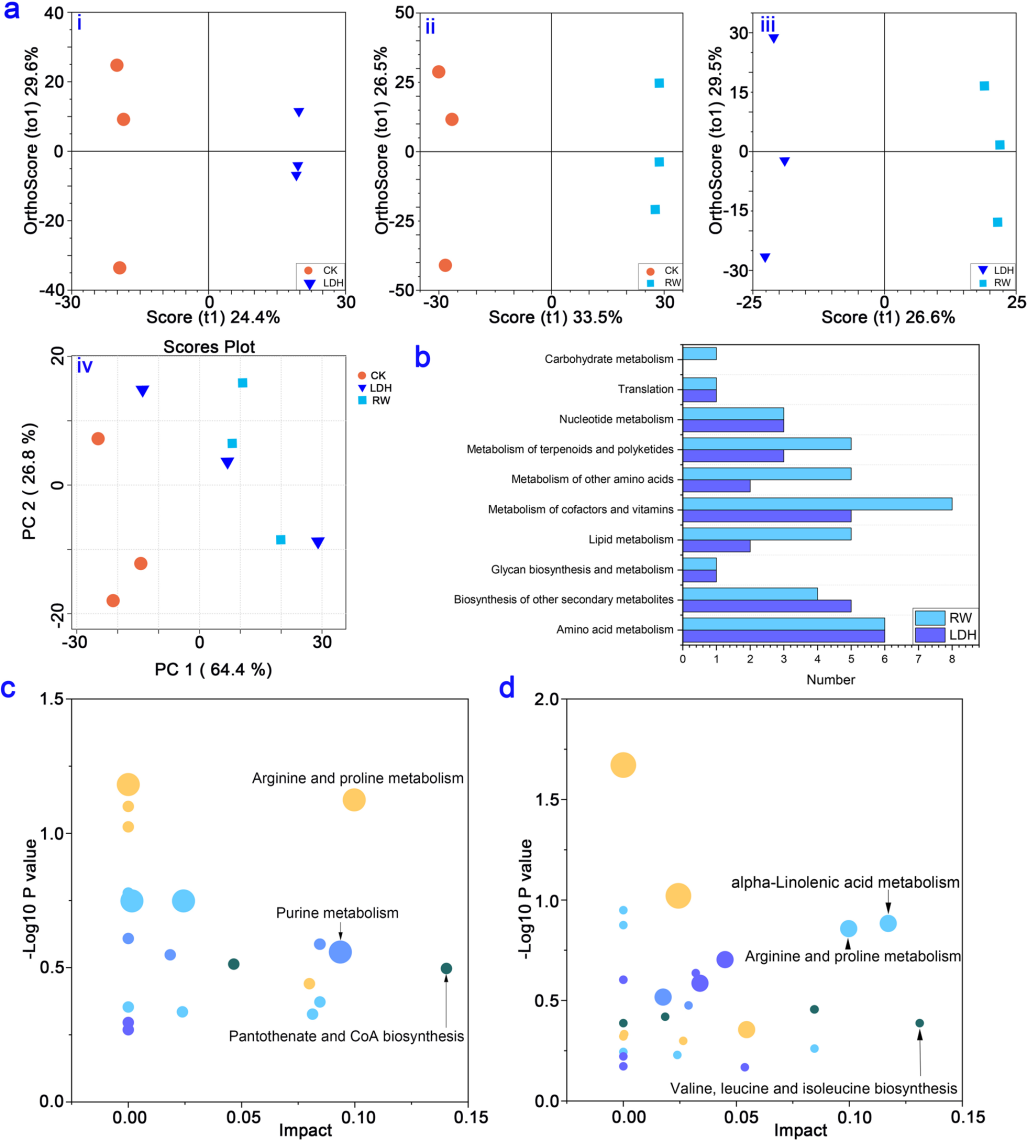
Metabolome analysis of lettuce leaves after 2 weeks of initial treatment. **a** Principal component analysis (PCA) plot and orthogonal partial least-squares discriminant analysis (OPLS-DA) model for identifying differential metabolites in the control and the LDH10 and RW10 treatments. LDH/CK (i), RW/CK (ii), LDH/RW (iii), and PCA (iv). The shape and color of the points correspond to different experimental groups. PC1: first principal component score; PC2: orthogonal principal component score; t1: first principal component score. **b** Annotation categories of the identified metabolites according to KEGG pathway analysis. Metabolic pathway analysis of the differential metabolites in the treatments of LDH10 **c** and RW10 **d** compared with the CK. The abscissa coordinate represents the value of the metabolic pathway. The bubble size indicates the number of metabolites. The vertical coordinate and bubble color indicate the p-value of the enrichment analysis. The abbreviations used are the same as in Fig. 2

By selecting MS peaks with orthogonal partial least-squares discriminant analysis (OPLS-DA) (VIP > 1, *p* < 0.05), the total numbers of differential metabolites in each treatment are shown in Additional file 1: Fig. S5. By employing principal component analysis (PCA) and OPLS-DA, we conducted an in-depth analysis of the distinctive metabolic changes stemming from LDH10 and RW10 exposure. The PCA score plot was used to assess the overall effect of LDH10 and RW10 on lettuce leaf metabolites (Fig. 5a). The sampling points corresponding to the three different treatments were noticeably scattered, indicating a substantial effect of LDH10 and RW10 on the metabolites of lettuce leaves. Notably, the effects of LDH10 and RW10 on the metabolic profiles differed significantly (Fig. 5a). Several metabolic pathways significantly differed following LDH10 or RW10 treatment relative to the control group. These pathways include carbohydrate metabolism, translation, nucleotide metabolism, metabolism of terpenoids and polyketides, metabolism of other amino acids, metabolism of cofactors and vitamins, lipid metabolism, glycan biosynthesis and metabolism, amino acid metabolism, and biosynthesis of other secondary metabolites (Fig. 5b). These findings highlight the prominent changes in various key metabolic processes as a result of LDH10 or RW10 treatment relative to the control group. The differential metabolites were analyzed using the Fisher tool to investigate changes in metabolic pathways. Comparative analysis revealed significant effects of the LDH10 or RW10 treatments on specific metabolic pathways compared with the CK (Fig. 5c, d). LDH10 treatment affected arginine and proline metabolism, purine metabolism, and pantothenate and CoA biosynthesis (Fig. 5c). RW10 treatment affected valine, leucine and isoleucine biosynthesis, arginine and proline metabolism, and alpha-linolenic acid metabolism (Fig. 5d). Arginine and proline metabolism was enriched in the leaves of all treatment groups, suggesting that it plays a key role in the response to both LDH10 and RW10 exposure.

Changes in gene expression are one of the various ways in which plants respond to external stimuli [59]. The environmental stress induced by the RW10 treatment was stronger than that induced by the LDH10 treatment, as indicated by the fact that a higher number of functional DEGs were detected in the RW10 treatment than in the LDH10 treatment (Fig. 4a, Additional file 1: Fig. S4). The regulation of gene expression led to changes in the metabolite profiles in lettuce leaves. The number of differential metabolites in lettuce leaves was higher in the RW10 treatment than in the LDH10 treatment (Additional file 1: Fig. S5). Moreover, the metabolic functions of lettuce leaves were significantly affected, especially under RW10-induced stress (Fig. 5). Both RW10 and LDH10 treatments led to the regulation of stress response-related DEGs in plants, including pathways such as plant-pathogen interaction and MAPK signaling pathway-plant (Fig. 4c). The expression of the genes associated with these pathways was up-regulated in profile 15 (Fig. 4c) following RW10 or LDH10 treatment, indicating that LDH10 potentially enhances the resilience of plants to stress. Similarly, Claudia Jonak et al. exposed the seedlings of Medicago plants to excessive copper or cadmium ions and found that they could activate the MAPK cascade reaction of higher plants, thereby reducing their toxicity [60]. The expression of genes related to mechanisms involved in photosynthesis, including the citrate cycle (TCA cycle), carbon metabolism, and photosynthesis, was up-regulated or down-regulated, suggesting that LDH10 and RW10 might induce imbalances in mechanisms related to photosynthesis (Fig. 4c). Zeatin, a well-known cytokinin plant hormone [61] and a regulator of plant growth [62], was elevated under stress conditions in various plant species. The expression of genes related to zeatin was higher in the LDH10 treatment than in the RW10 treatment, indicating that the up-regulation of zeatin could potentially mediate the response of lettuce to LDH10 (Profile 15, Fig. 4b, c). The expression of the genes in Profile 12, 13, and 15 was up-regulated in the LDH10 and RW10 treatments relative to the CK, and the expression of these genes was significantly up-regulated in the LDH10 treatment relative to the RW10 treatment; these genes are associated with the plant-pathogen interaction and MAPK signaling pathway-plant pathways. The plant-pathogen interaction [63] and MAPK signaling pathway-plant [60] are associated with stress resistance, suggesting that LDH10 may enhance the ability of plants to adapt to stress and induce the expression of stress resistance genes compared with the RW10 treatment. The above data indicate that the stress resistance capacity was higher in the LDH10 treatment than in the RW10 treatment.

**Fig. 6:**
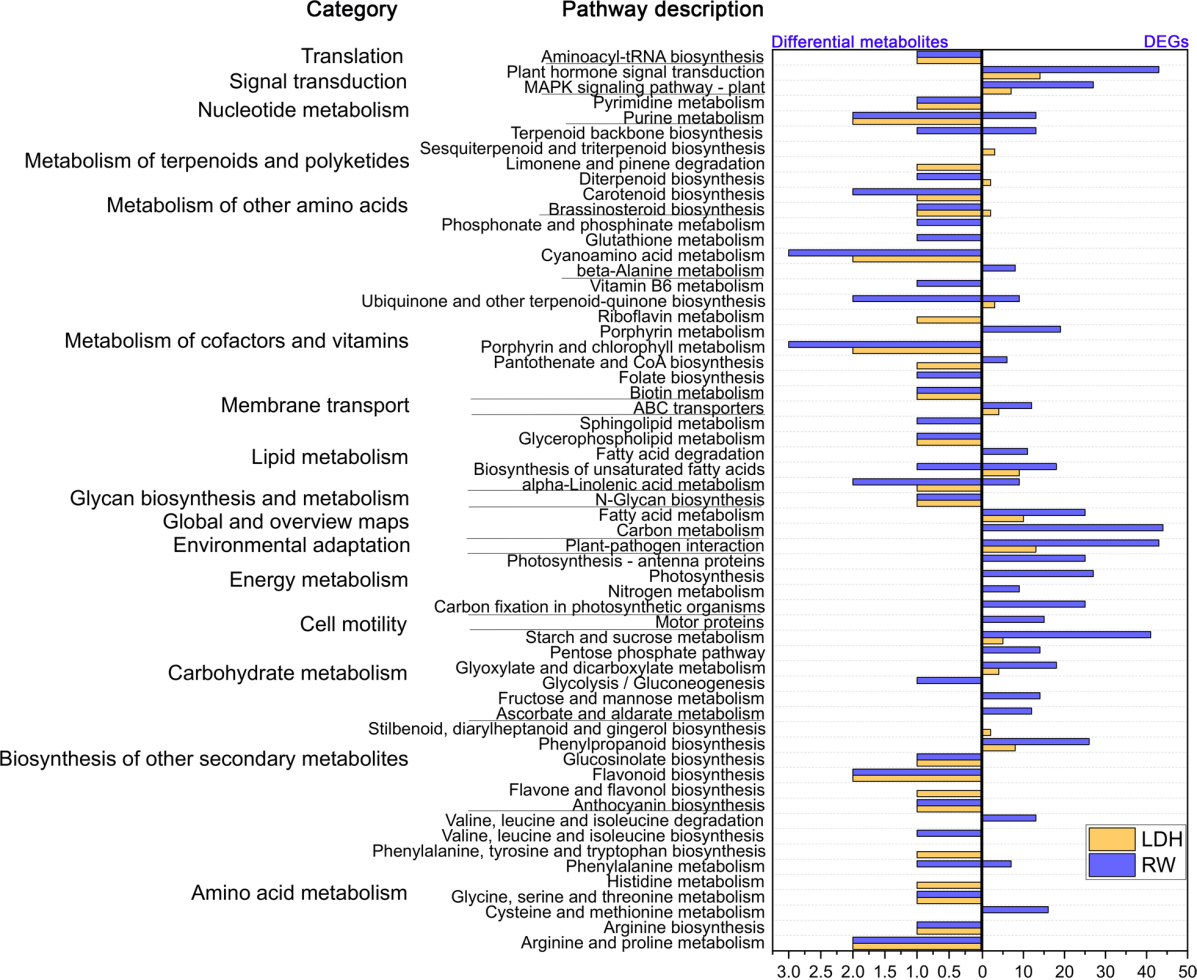
Integration of DEGs and differential metabolites with KEGG pathway annotations following the LDH10 and RW10 treatments. Abbreviations are the same as in Fig. 2

The combined analysis of DEGs and differential metabolites did not reveal shared regulatory mechanisms between LDH10 and RW10 (Fig. 6). RW10 regulated purine metabolism, terpenoid backbone biosynthesis, ubiquinone and other terpenoid-quinone biosynthesis, alpha-linolenic acid metabolism, and biosynthesis of unsaturated fatty acids (Fig. 6). Purine metabolism is a key regulated metabolic pathway in Arabidopsis under drought stress and in rice under spaceflight stress; it also plays a significant role in the ability of rice seedlings to tolerate darkness [64]. The terpenoid backbone biosynthesis pathway and the biosynthesis of ubiquinone and other terpenoid-quinone compounds are responsible for the synthesis of various terpenoids and ubiquinones, which affect multiple physiological functions [65]. Alpha-linolenic acid metabolism and the biosynthesis of unsaturated fatty acids play a role in increasing the content of unsaturated fatty acids to counteract the loss of cellular membrane fluidity induced by adverse conditions [66]. Significantly, LDH10 induced the biosynthesis of Brassinosteroids (BR) (Fig. 6). In light of the known role of BR in promoting growth and enhancing stress resistance [67], LDH10 promoted the expression of genes involved in the biosynthesis of BR, which led to the increased production of BR. These findings regarding the effect on hormonal changes are consistent with previous studies that demonstrated alterations in auxin content and flux in Arabidopsis roots by MgAl-LDHs [16]. Furthermore, the qRT-PCR results (Additional file 1: Fig. S6) revealed that the expression of genes involved in the synthesis of BR was up-regulated following LDH treatment, suggesting that this could be the primary molecular mechanism by which LDH promotes growth.

## Conclusions

The results of this study make a significant contribution to our understanding of the effect of CuFe-LDHs on the growth and physiological state of lettuce. Our findings also provide valuable insights into the molecular mechanisms underlying the biological effects of CuFe-LDHs on leafy vegetables. In general, our results have the potential to enhance our understanding of cell fate and the effects of CuFe-LDHs, which would facilitate the development of nanofertilizers containing CuFe-LDHs.

### Supplementary Information

**Additional file 1: Section S1**. Transcriptome analysis of lettuce. **Section S2**. Metabolomics analysis of lettuce. **Figure S1**. Characterization of CuFe-LDHs by using TEM. **Figure S2**. Zeta potential of CuFe-LDHs by using Dynamic Light Scattering (DLS). **Figure S3**. The particle size of of CuFe-LDHs by using DLS. **Figure S4**. Venn diagram of differentially expressed genes (DEGs) between different comparison groups. **Figure S5**. Venn diagram of differential metabolites with OPLS-DA (VIP > 1, *p* < 0.05) among different treatment groups. **Figure S6**. qRT-PCR. **Table S1**. The formulation of modified Yamazaki nutrient solution. **Table S2**. Primer sequences for qRT-PCR. **Table S3**. Summary of the sequencing reads and read mapping.

## References

[1] Fawzy ZF, El-Sawy SM, El-Bassiony AE-M, Jun H, Shedeed SI, Okasha AM, Bayoumi Y, El-Ramady H, Prokisch J (2022). Smart fertilizers vs nano-fertilizers: a pictorial overview. Env Biodiv Soil Sec.

[2] Jiang M, Song Y, Kanwar MK, Ahammed GJ, Shao S, Zhou J (2021). Phytonanotechnology applications in modern agriculture. J Nanobiotechnol.

[3] Lowry GV, Avellan A, Gilbertson LM (2019). Opportunities and challenges for nanotechnology in the agri-tech revolution. Nat Nanotechnol.

[4] Sharma B, Tiwari S, Kumawat KC, Cardinale M (2023). Nano-biofertilizers as bio-emerging strategies for sustainable agriculture development: potentiality and their limitations. Sci Total Environ.

[5] An CC, Sun CJ, Li NJ, Huang BN, Jiang JJ, Shen Y, Wang C, Zhao X, Cui B, Wang CX, Li XY, Zhan SS, Gao F, Zeng ZH, Cui HX, Wang Y (2022). Nanomaterials and nanotechnology for the delivery of agrochemicals: strategies towards sustainable agriculture. J Nanobiotechnol.

[6] Giri VP, Shukla P, Tripathi A, Verma P, Kumar N, Pandey S, Dimkpa CO, Mishra A (2023). A review of sustainable use of biogenic nanoscale agro-materials to enhance stress tolerance and nutritional value of plants. Plants.

[7] Bhardwaj AK, Arya G, Kumar R, Hamed L, Pirasteh-Anosheh H, Jasrotia P, Kashyap PL, Singh GP (2022). Switching to nanonutrients for sustaining agroecosystems and environment: the challenges and benefits in moving up from ionic to particle feeding. J Nanobiotechnol.

[8] Zhu ZX, Shao CH, Guo YL, Feng JG, Chen C, Yang HB (2021). Facile pathway to generate agrochemical nanosuspensions integrating super-high load, eco-friendly excipients, intensified preparation process, and enhanced potency. Nano Res.

[9] Liu RQ, Lal R (2015). Potentials of engineered nanoparticles as fertilizers for increasing agronomic productions. Sci Total Environ.

[10] Dietz KJ, Herth S (2011). Plant nanotoxicology. Trends Plant Sci.

[11] Santos AR, Miguel AS, Tomaz L, Malhó R, Maycock C, Patto MCV, Fevereiro P, Oliva A (2010). The impact of CdSe/ZnS quantum dots in cells of Medicago sativa in suspension culture. J Nanobiotechnol.

[12] Husted S, Minutello F, Pinna A, Tougaard SL, Mos P, Kopittke PM (2023). What is missing to advance foliar fertilization using nanotechnology?. Trends Plant Sci.

[13] Husen A, Siddiqi KS (2014). Carbon and fullerene nanomaterials in plant system. J Nanobiotechnol.

[14] Garza-Alonso CA, Juárez-Maldonado A, González-Morales S, Cabrera-De la Fuente M, Cadenas-Pliego G, Morales-Díaz AB, Trejo-Téllez LI, Tortella G, Benavides-Mendoza A. ZnO nanoparticles as potential fertilizer and biostimulant for lettuce. Heliyon. 2023;9:e12787. 10.1016/j.heliyon.2022.e12787 PMC9840361 36647345

[15] Mu QL, Chen KJ, Li YH, Li TQ (2023). Effects of zinc oxide nanoparticles on nutrient uptake and photosynthesis of lettuce. J Zhejiang Univ.

[16] Wu HY, Zhang H, Li XY, Zhang Y, Wang JK, Wang Q, Wan YL (2022). Optimized synthesis of layered double hydroxide lactate nanosheets and their biological effects on Arabidopsis seedlings. Plant Methods.

[17] Yousefi V, Tarhriz V, Eyvazi S, Dilmaghani A (2020). Synthesis and application of magnetic@layered double hydroxide as an anti-inflammatory drugs nanocarrier. J Nanobiotechnol.

[18] Zhao JY, Wu H, Zhao J, Yin YL, Zhang ZL, Wang SG, Lin K (2021). 2D LDH-MoS(2) clay nanosheets: synthesis, catalase-mimic capacity, and imaging-guided tumor photo-therapy. J Nanobiotechnol.

[19] Yu J, Wang Q, O'Hare D, Sun L (2017). Preparation of two dimensional layered double hydroxide nanosheets and their applications. Chem Soc Rev.

[20] Bao WL, Wang JY, Wang Q, O'Hare D, Wan YL (2016). Layered double hydroxide nanotransporter for molecule delivery to intact plant cells. Sci Rep.

[21] Wang JY, Bao WL, Umar A, Wang Q, O'Hare D, Wan YL (2016). Delaminated layered double hydroxide nanosheets as an efficient vector for DNA delivery. J Biomed Nanotechnol.

[22] Song YP, Xuan AR, Bu CH, Ci D, Tian M, Zhang DQ (2019). Osmotic stress-responsive promoter upstream transcripts (PROMPTs) act as carriers of MYB transcription factors to induce the expression of target genes in Populus simonii. Plant Biotechnol J.

[23] Zhang H, Liu S, Li XY, Yao L, Wu HY, Baluška F, Wan YL (2021). An antisense circular RNA regulates expression of RuBisCO small subunit genes in Arabidopsis. Front Plant Sci.

[24] Velázquez-Herrera FD, Lobo-Sánchez M, Fetter G (2022). LDH/SBA-15 nanocomposite containing nitrogen-fixing bacteria as an efficient biofertilizer. Mater Today Commun.

[25] Bao WL, Wan YL, Baluska F (2017). Nanosheets for delivery of biomolecules into plant cells. Trends Plant Sci.

[26] Li Y, Bao WL, Wu HY, Wang JY, Zhang Y, Wan YL, Cao DP, O'Hare D, Wang Q (2017). Delaminated layered double hydroxide delivers DNA molecules as sandwich nanostructure into cells via a non-endocytic pathway. Sci Bull.

[27] Mitter N, Worrall EA, Robinson KE, Li P, Jain RG, Taochy C, Fletcher SJ, Carroll BJ, Lu GQ, Xu ZP (2017). Clay nanosheets for topical delivery of RNAi for sustained protection against plant viruses. Nat Plants.

[28] Liu QL, Li YP, Xu KD, Li DX, Hu HY, Zhou F, Song PW, Yu YG, Wei QC, Liu Q, Wang WP, Bu RF, Sun HL, Wang XH, Hao JJ, Li HL, Li CW (2020). Clay nanosheet-mediated delivery of recombinant plasmids expressing artificial miRNAs via leaf spray to prevent infection by plant DNA viruses. Hortic Res.

[29] Jain RG, Fletcher SJ, Manzie N, Robinson KE, Li P, Lu E, Brosnan CA, Xu ZP, Mitter N (2022). Foliar application of clay-delivered RNA interference for whitefly control. Nat Plants.

[30] Palmer CM, Guerinot ML (2009). Facing the challenges of Cu, Fe and Zn homeostasis in plants. Nat Chem Biol.

[31] Abadía J, López-Millán A, Rombolà A, Abadía A (2002). Organic acids and Fe deficiency: a review. Plant Soil.

[32] Rahman N, Schoenau J (2020). Response of wheat, pea, and canola to micronutrient fertilization on five contrasting prairie soils. Sci Rep.

[33] Gawande MB, Goswami A, Felpin FX, Asefa T, Huang X, Silva R, Zou X, Zboril R, Varma RS (2016). Cu and Cu-Based nanoparticles: synthesis and applications in catalysis. Chem Rev.

[34] Laipan M, Fu H, Zhu R, Sun L, Zhu J, He H (2017). Converting spent Cu/Fe layered double hydroxide into Cr(VI) reductant and porous carbon material. Sci Rep.

[35] Liu HB, Jiao QZ, Zhao Y, Li HS, Sun CB, Li XF, Wu HY (2010). Cu/Fe hydrotalcite derived mixed oxides as new catalyst for thermal decomposition of ammonium perchlorate. Mater Lett.

[36] Schwenk CF, Rode BM (2003). The influence of the Jahn-Teller effect and of heteroligands on the reactivity of Cu^2+^. Chem Commun.

[37] Liu RQ, Zhang HY, Lal R (2016). Effects of stabilized nanoparticles of copper, zinc, manganese, and iron oxides in low concentrations on lettuce (*Lactuca sativa*) seed germination: nanotoxicants or nanonutrients?. Water Air Soil Poll.

[38] Nair PMG, Chung IM (2014). Impact of copper oxide nanoparticles exposure on Arabidopsis thaliana growth, root system development, root lignificaion, and molecular level changes. Environ Sci Pollut R.

[39] Holloway PJ (1969). The effects of superficial wax on leaf wettability. Ann Appl Biol.

[40] Ellis MC, Webb DA, Western NM (2004). The effect of different spray liquids on the foliar retention of agricultural sprays by wheat plants in a canopy. Pest Manag Sci.

[41] Gu Z, Li S, Zhang F, Wang S (2016). Understanding surface adhesion in nature: a peeling model. Adv Sci.

[42] McGrane SJ, Mainwaring DE, Cornell HJ, Rix CJ (2004). The role of hydrogen bonding in amylose gelation. Starch-Stärke.

[43] Herschlag D, Pinney MM (2018). Hydrogen bonds: simple after all?. Biochemistry.

[44] Matsumoto K, Tsubaki D, Sekine K, Kubota H, Minamiya K, Yamanaka S (2017). Influences of number of hydroxyl groups and cooling solid surface temperature on ice adhesion force. Inter J Refrig.

[45] Wang Y, Xiang LL, Wang F, Wang ZQ, Bian YR, Gu CG, Wen X, Kengara FO, Schaffer A, Jiang X, Xing B (2022). Positively charged microplastics induce strong lettuce stress responses from physiological, transcriptomic, and metabolomic perspectives. Environ Sci Technol.

[46] Choy J, Choi S, Oh J, Park T (2007). Clay minerals and layered double hydroxides for novel biological applications. Appl Clay Sci.

[47] Yruela I (2009). Copper in plants: acquisition, transport and interactions. Funct Plant Biol.

[48] Barr R, Crane FL (1976). Organization of electron transport in photosystem II of spinach chloroplasts according to chelator inhibition sites. Plant Physiol.

[49] Krohling CA, Eutrópio FJ, Bertolazi AA, Dobbss LB, Campostrini E, Dias T, Ramos AC (2016). Ecophysiology of iron homeostasis in plants. Soil Sci Plant Nutr.

[50] Behrenfeld MJ, Bale AJ, Kolber ZS, Aiken J, Falkowski PG (1996). Confirmation of iron limitation of phytoplankton photosynthesis in the equatorial Pacific Ocean. Nature.

[51] Briat JF, Curie C, Gaymard F (2007). Iron utilization and metabolism in plants. Curr Opin Plant Biol.

[52] Pereira EG, Oliva MA, Rosado-Souza L, Mendesa GC, Colares DS, Stopato CH, Almeida AM (2013). Iron excess affects rice photosynthesis through stomatal and non-stomatal limitations. Plant Sci.

[53] Datt B (1998). Remote sensing of chlorophyll *a*, chlorophyll* b*, chlorophyll *a*+*b*, and total carotenoid content in eucalyptus leaves. Remote Sens Environ.

[54] Mardani HR (2017). (Cu/Ni)–Al layered double hydroxides@Fe_3_O_4_ as efficient magnetic nanocomposite photocatalyst for visible-light degradation of methylene blue. Res Chem Intermed.

[55] Gupta AS, Webb RP, Holaday AS, Allen RD (1993). Overexpression of superoxide-dismutase protects plants from oxidative stress - induction of ascorbate peroxidase in superoxide dismutase-overexpressing plants. Plant Physiol.

[56] Liu J, Hu XT, Wang WE, Ran H, Fang SL, Yang X (2019). Resoinse of growth, quality and element utilization efficiency of hydroponic lettuce to light intensity and photoperiod. N Hortic.

[57] Tian D, Ren YF, Wang YL, Lin X, Yang B, He JY (2018). Effects of cadmium stress on seed germination, seedling growth and antioxidant enzyme system of lettuce. N Hortic.

[58] Ma SS, Xin JP, Tian RN (2020). Photosynthetic adaptability of pontederia cordata to copper stress. J Ne For Univ.

[59] Cattivelli L, Baldi P, Crosatti C, Fonzo ND, Faccioli P, Grossi M, Mastrangelo AM, Pecchioni N, Stanca AM (2002). Chromosome regions and stress-related sequences involved in resistance to abiotic stress in Triticeae. Plant Mol Biol.

[60] Nakagami H, Pitzschke A, Hirt H (2005). Emerging MAP kinase pathways in plant stress signalling. Trends Plant Sci.

[61] Anfang M, Shani E (2021). Transport mechanisms of plant hormones. Curr Opin Plant Biol.

[62] Schäfer M, Brütting C, Meza-Canales ID, Großkinsky DK, Vankova R, Baldwin IT, Meldau S (2015). The role of *cis*-zeatin-type cytokinins in plant growth regulation and mediating responses to environmental interactions. J Exp Bot.

[63] Kahlon PS, Stam R (2021). Polymorphisms in plants to restrict losses to pathogens: from gene family expansions to complex network evolution. Curr Opin Plant Biol.

[64] Watanabe S, Nakagawa A, Izumi S, Shimada H, Sakamoto A (2010). RNA interference-mediated suppression of xanthine dehydrogenase reveals the role of purine metabolism in drought tolerance in Arabidopsis. FEBS Lett.

[65] Sun ZJ, Li ZX (2018). The terpenoid backbone biosynthesis pathway directly affects the biosynthesis of alarm pheromone in the aphid. Insect Mol Biol.

[66] Mata-Pérez C, Sánchez-Calvo B, Begara-Morales JC, Luque F, Jiménez-Ruiz J, Padilla MN, Fierro-Risco J, Valderrama R, Fernández-Ocaña A, Corpas FJ, Barroso JB (2015). Transcriptomic profiling of linolenic acid-responsive genes in ROS signaling from RNA-seq data in Arabidopsis. Front Plant Sci.

[67] Clouse SD, Sasse JM (1998). Brassinosteroids: essential regulators of plant growth and development. Annu Rev Plant Physiol Plant Mol Biol.

