# Supplementary material for: Enhancing lettuce yield via Cu/Fe-layered double hydroxide nanoparticles spraying
Source: J Nanobiotechnology. 2023 Nov 11;21:417. doi: 10.1186/s12951-023-02178-6 (PMC10638715; doi:10.1186/s12951-023-02178-6)
Supplement: Supplementary file 1 — Additional file 1: Section S1. Transcriptome analysis of lettuce. Section S2. Metabolomics analysis of lettuce. Figure S1. Characterization of CuFe-LDHs by using TEM. Figure S2. Zeta potential of CuFe-LDHs by using Dynamic Light Scattering (DLS). Figure S3. The particle size of of CuFe-LDHs by using DLS. Figure S4. Venn diagram of differentially expressed genes (DEGs) between different comparison groups. Figure S5. Venn diagram of differential metabolites with OPLS-DA (VIP > 1, p < 0.05) among different treatment groups. Figure S6. qRT-PCR. Table S1. The formulation of modified Yamazaki nutrient solution. Table S2. Primer sequences for qRT-PCR. Table S3. Summary of the sequencing reads and read mapping. [file 12951_2023_2178_MOESM1_ESM.docx]

**Supporting information**

**Enhancing lettuce yield via Cu/Fe-layered double hydroxide nanoparticles spraying**

*Hongyang Wu^1‡^, Xiaoyang Wan^1‡^, Jiefei Niu^3^, Huimin Xu^4^, Yu Zhang^5^, Xian Xue^6^, Yang Li^1^, Qiang Li^1^, Tao Lu^1^, Hongjun Yu^1^*, and Weijie Jiang^12^**

^1^ State Key Laboratory of Vegetable Biobreeding, Institute of Vegetables and Flowers, Chinese Academy of Agricultural Sciences, Beijing 100081, China.

^2^ College of Horticulture, Xinjiang Agricultural University, Urumqi 830052, China.

^3^ Research Unit of Molecular Epidemiology, Helmholtz Zentrum München, Neuherberg 85764, Germany.

^4^ College of Biological Sciences, China Agricultural University, Beijing 100193, China. ^5^ Faculty of Environmental Science and Engineering, Kunming University of Science and Technology, Kunming 650500, China.

^6^ College of Agriculture, Henan University of Science and Technology, Luoyang 471000, China.

**Keywords:** Layered double hydroxides, Lettuce, Phenotypic analysis

*Corresponding author at: State Key Laboratory of Vegetable Biobreeding, Institute of Vegetables and Flowers, Chinese Academy of Agricultural Sciences, Beijing 100081, P.R. China. E-mail address: jiangweijie@caas.cn

^‡^ These authors contributed equally to this work.

**Additional file1:**

**Section S1. Transcriptome analysis of lettuce**

**S1.1.** **The experimental workflow**

The experimental workflow involved several steps: total RNA extraction, mRNA enrichment using Oligo dT, mRNA fragmentation, reverse transcription to generate cDNA, adaptor ligation, and sequencing on the Illumina platform. Tissue samples were used for total RNA extraction, and the Nanodrop 2000 (Thermo Fisher Scientific, USA) was used to evaluate the RNA concentration and purity. RNA integrity was assessed through agarose gel electrophoresis, and the RNA Integrity Number (RIN) was determined using the Agilent 2100 system (Agilent Technologies, USA). For each library preparation, a minimum of 1 μg of total RNA was needed, with a concentration of at least 35 ng/μL, an OD260/280 ratio ≥1.8, and an OD260/230 ratio ≥1.0. In eukaryotes, mRNA molecules feature a poly-A tail at their 3' end. To selectively isolate mRNA from total RNA for transcriptomic analysis, magnetic beads coated with Oligo(dT) were used due to their specific binding to the poly-A tail. The Illumina Novaseq 6000 platform (Illumina, USA) was chosen for sequencing short sequence fragments. The isolated mRNA, consisting of full-length RNA sequences averaging several kilobases in length, required fragmentation. This was achieved by introducing a fragmentation buffer, causing random fragmentation of the mRNA. Magnetic bead selection was then used to isolate small fragments approximately 300 bp in size. During reverse transcription, a random hexamer primer was added to initiate the synthesis of first-strand cDNA using the mRNA template. Subsequently, second-strand synthesis was performed, resulting in the formation of stable double-stranded cDNA. PCR amplification was conducted for 15 cycles. Following amplification, libraries underwent size selection using a 2% Agarose gel to isolate cDNA fragments measuring 300 bp in length. Library quantification was performed using the TBS380 assay (Invitrogen, USA). Bridge PCR amplification was conducted on the cBot instrument to generate clusters. Paired-end RNA sequencing was performed on a sequencer with a read length of 2 × 150 bp.

**S1.2.** **Read mapping**

The Table S3 shows the reads, guanine-cytosine percentage (GC%), and average quality score of the sequenced data. The accuracy and quality of these data, along with the mapping efficiency to the lettuce (*Lactuca sativa* L.) genome and the unique mapping rates, are sufficient for further analysis. To verify the RNA-Seq results, four differentially expressed genes (DEGs) were randomly selected, and their gene expression was detected using real-time quantitative PCR (qPCR). The log2FC and variation trends of all the selected genes observed in the qPCR analysis were consistent with the RNA-Seq findings. This result confirms the validity of RNA sequencing and its suitability for subsequent analysis.

**S1.3. Differential expression analysis and functional enrichment**

The expression level of each gene was determined using the transcripts per million reads (TPM) method to identify differential expression genes (DEGs) between two samples. RSEM (http://deweylab.biostat.wisc.edu/rsem/) was used to quantify gene abundances. Differential expression analysis was performed using DESeq24, with a p-adjust value ≤ 0.05 and |log2FC|>2 as the criteria for significance. Additionally, KOBAS (http://kobas.cbi.pku.edu.cn/home.do) was utilized to conduct Kyoto Encyclopedia of Genes and Genomes (KEGG) pathway analysis.

**Section S2.** **Metabolomics analysis of lettuce**

**S2.1. Metabolites Extraction**

The LC/MS system used for metabolomics analysis consists of the Waters Acquity I-Class PLUS ultra-high-performance liquid chromatography and the Waters Xevo G2-XS QTof high-resolution mass spectrometer. The column utilized is the Waters Acquity UPLC HSS T3 column (1.8um 2.1*100mm). In positive ion mode, the mobile phase consists of a 0.1% formic acid aqueous solution (mobile phase A) and 0.1% formic acid acetonitrile (mobile phase B). In negative ion mode, the mobile phase remains the same. The injection volume is 1μL.

**S2.2. LC-MS/MS Analysis**

The Waters Xevo G2-XS QTOF high-resolution mass spectrometer, controlled by the MassLynx V4.2 acquisition software from Waters, is capable of collecting primary and secondary mass spectrometry data in MSe mode. During each data acquisition cycle, dual-channel data acquisition is performed simultaneously at both low collision energy (2V) and a range of high collision energy (10~40V). The scanning frequency for obtaining a mass spectrum is 0.2 seconds.

The parameters of the ESI ion source are as follows:

- Capillary voltage: 2000V (positive ion mode) or -1500V (negative ion mode)
- Cone voltage: 30V
- Ion source temperature: 150°C
- Desolvent gas temperature: 500°C
- Backflush gas flow rate: 50L/h
- Desolventizing gas flow rate: 800L/h.

**S2.3. Data preprocessing and annotation**

The raw data obtained through MassLynx V4.2 is processed using Progenesis QI software for peak extraction, alignment, and other data processing tasks. This software utilizes the Progenesis QI online METLIN database and Biomark's proprietary library for identification. Moreover, it ensures that theoretical fragment identification and mass deviation are kept within 100ppm.

## Figure


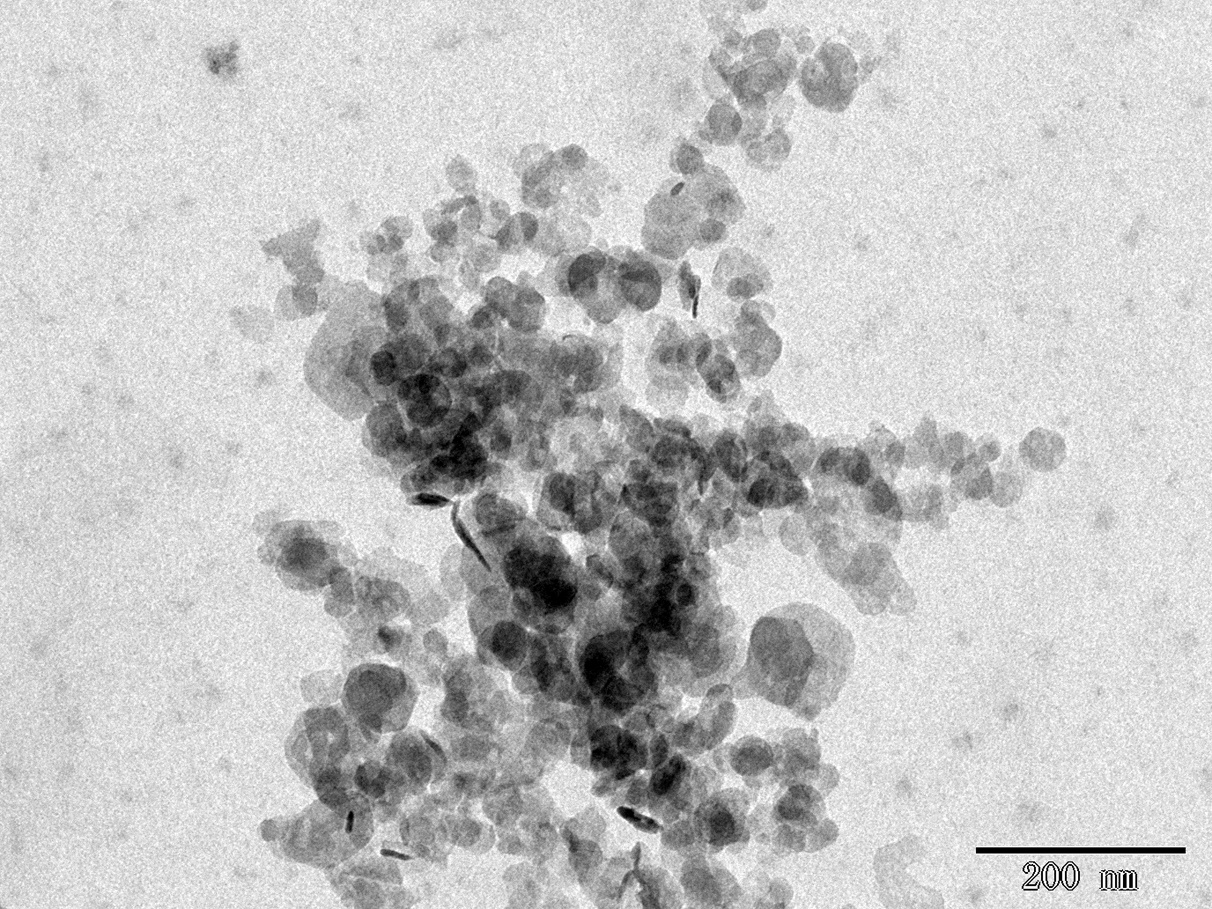


**Figure S1** Characterization of CuFe-LDHs by using TEM


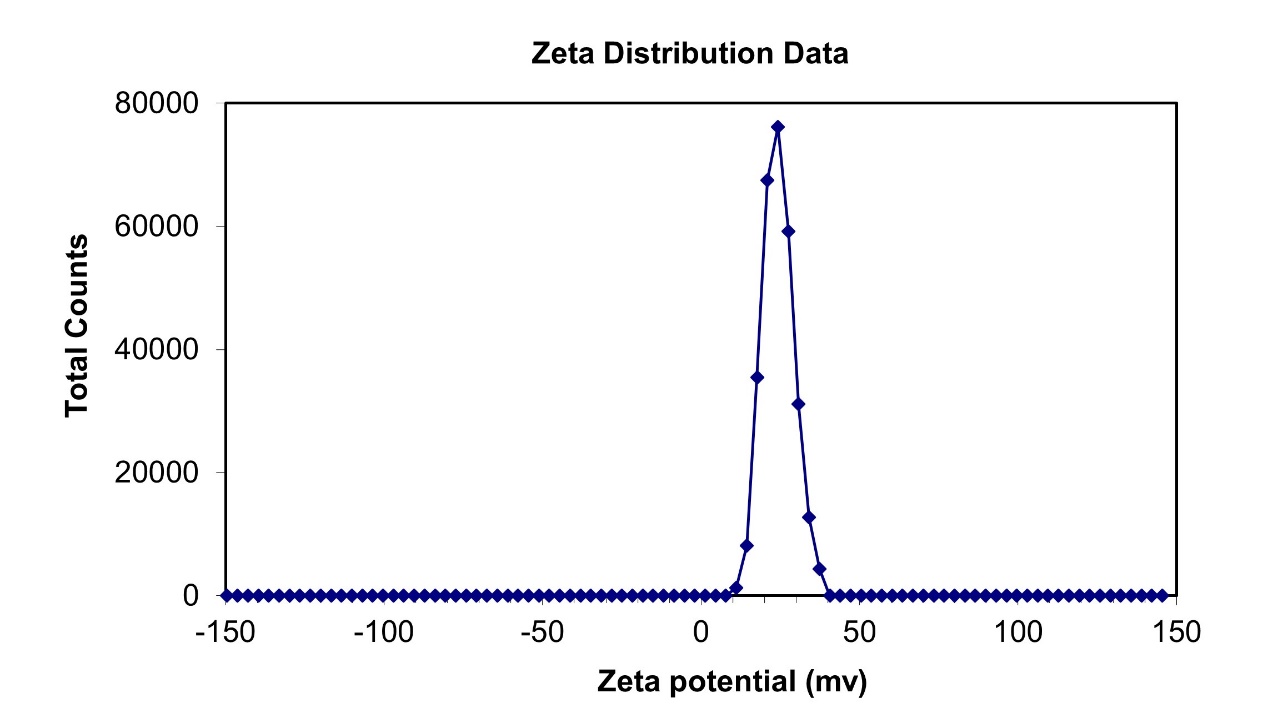


**Figure S2** Zeta potential of CuFe-LDHs by using Dynamic Light Scattering (DLS)


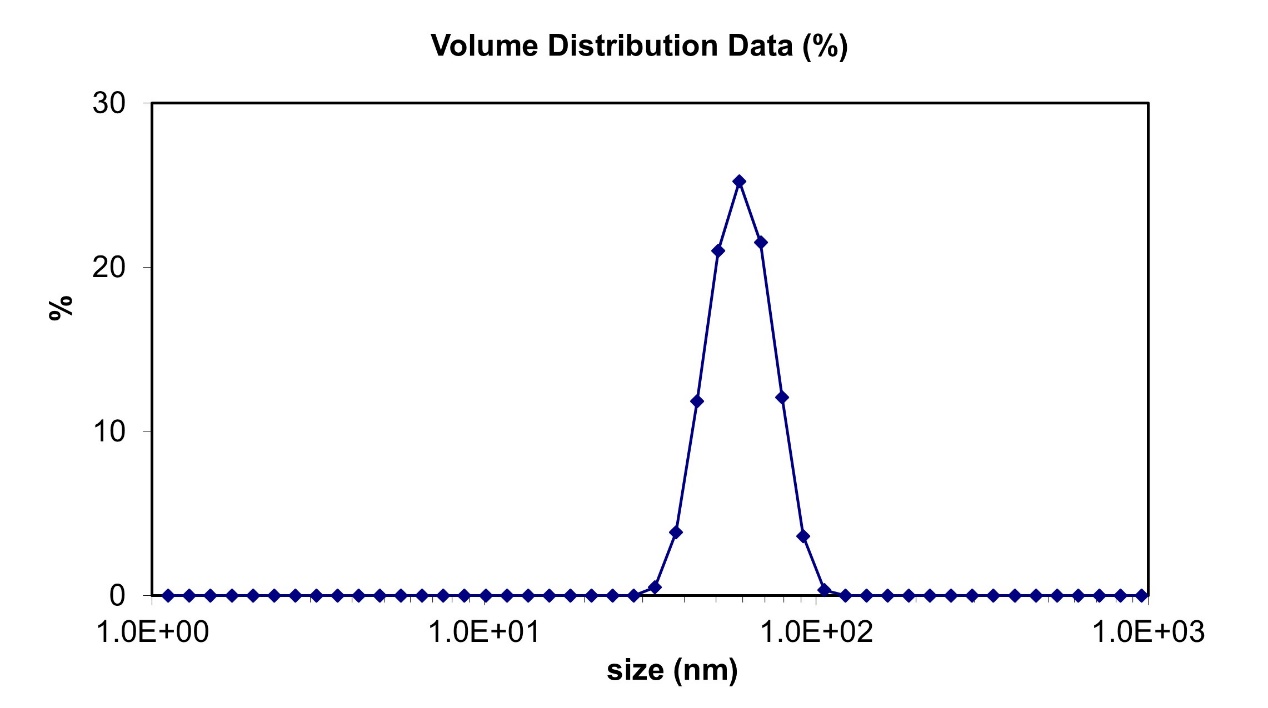


**Figure S3** The particle size of of CuFe-LDHs by using DLS


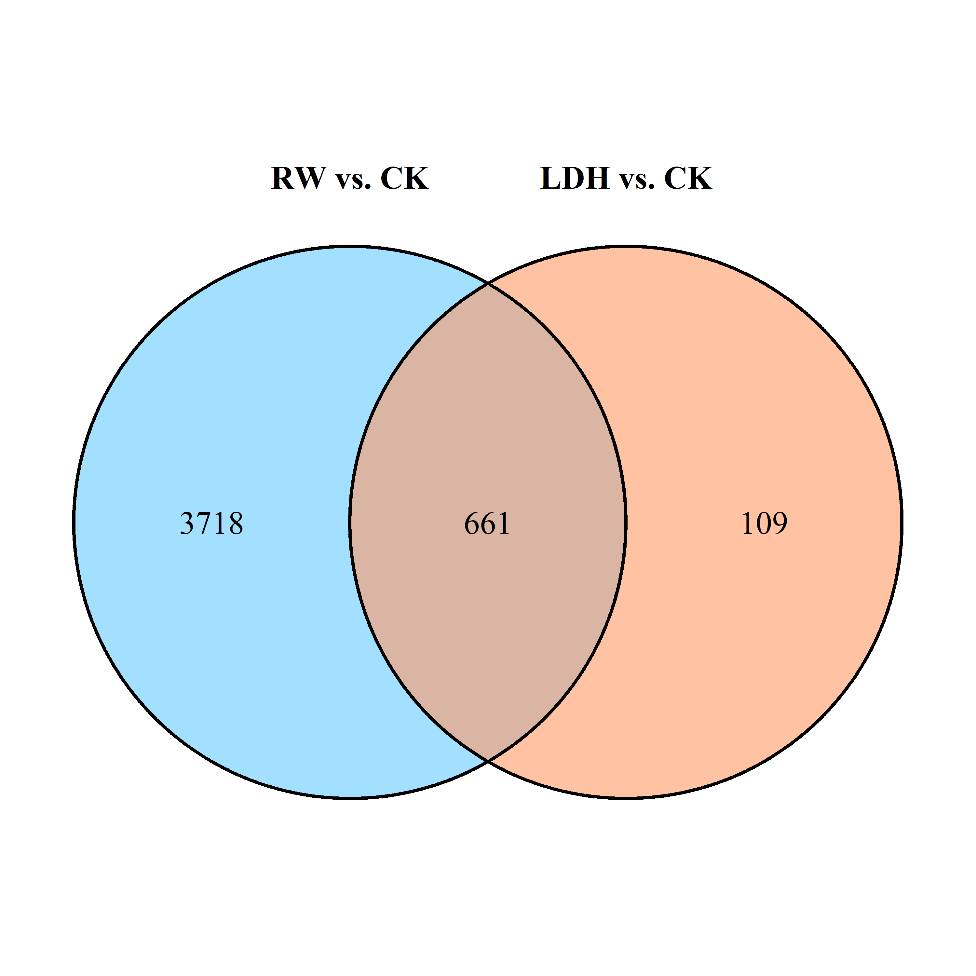


**Figure S4** Venn diagram of differentially expressed genes (DEGs) between different comparison groups. Genes/transcripts that exhibited a false discovery rate (FDR) below 0.05 and an absolute fold change ≥ 2 compared to the control (CK) were regarded as differentially expressed genes (DEGs).

**
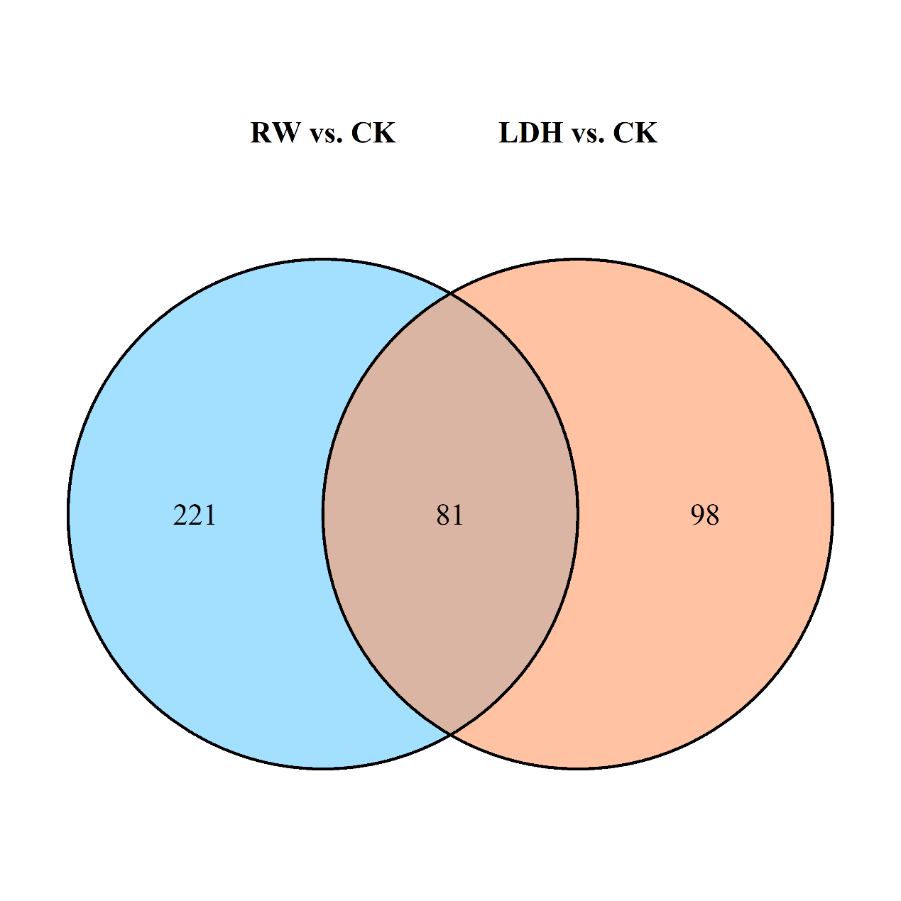
**

**Figure S5** Venn diagram of differential metabolites with OPLS-DA (VIP > 1, *p* < 0.05) among different treatment groups


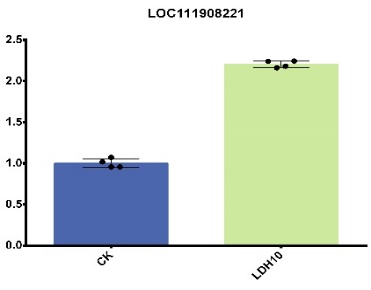

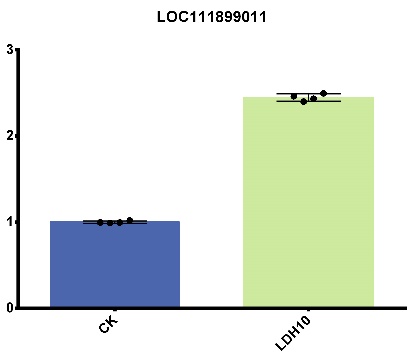

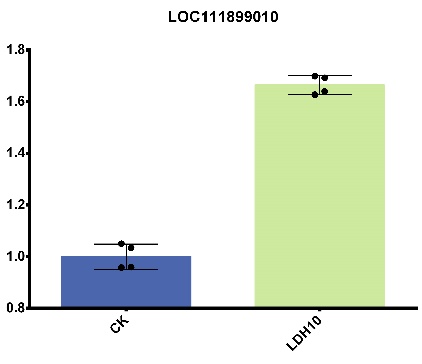

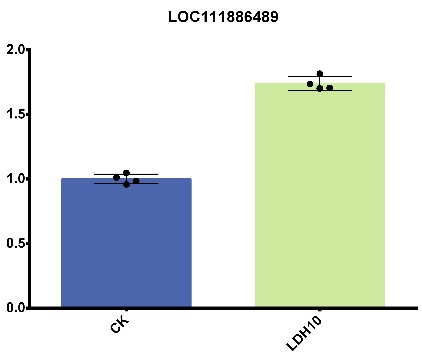

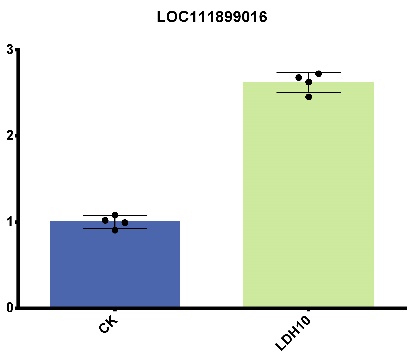


**Figure S6** qRT-PCR. LOC111908221 BRASSINOSTEROID INSENSITIVE 1-associated receptor kinase 1 (BAK1) LOC111899016 BRASSINOSTEROID INSENSITIVE 1-associated receptor kinase 1, transcript variant X1; LOC111886489 brassinosteroid-responsive RING protein 1; LOC111899011 BRASSINOSTEROID INSENSITIVE 1-associated receptor kinase 1(BAK1); LOC111899010 BRASSINOSTEROID INSENSITIVE 1-associated receptor kinase 1(BAK1)

## Table

**Table S1** The formulation of modified Yamazaki nutrient solution

| **Macronutrients** | **Concentration (mg/L)** | **Micronutrients** | **Concentration (mg/L)** |
| --- | --- | --- | --- |
| Ca(NO_3_)_2_•4H_2_O | 826 | H_3_BO_3_ | 4.5 |
| KNO_3_ | 607 | FeSO_4_ | 2.86 |
| MgSO_4_•7H_2_O | 483 | MnSO_4_•4H_2_O | 2.13 |
| NH_4_H_2_PO_4_ | 115 | ZnSO_4_•7H_2_O | 0.22 |
|  |  | CuSO_4_•5H_2_O | 0.08 |
|  |  | (NH_4_)_2_•4H_2_OMo_7_O_24_ | 0.02 |
|  |  | NaFe-EDTA | 20 |

**Table S2** Primer sequences for qRT-PCR

| **Gene id** | **Forward primer (5'-3')** | **Reverse Primer(5'-3')** |
| --- | --- | --- |
| LOC111908221 | TGAAAGCTCCCGCAGTTTCT | TCCGGATCCTCTTCGGCTAA |
| LOC111899016 | CGGGTTGGTGCGTCAAAAAT | GTTGTTTTCTGTCCCGCCAC |
| LOC111886489 | CGTCACCGTGTTTCCACCTA | GTCGGGGATTAACGGAGTCC |
| LOC111899011 | GAATCTGCTTCGGCTGCAAG | GCCCAAGATCATAGGCACGT |
| LOC111899010 | TACATGGCCAATGGAAGCGT | TTGTCCCACGTACAGCTGTC |

**Table S3** Summary of the sequencing reads and read mapping

| **Sample** | **Raw reads** | **Raw bases** | **Clean reads** | **Clean Bases** | **Error%** | **Q20%** | **Q30%** | **GC%** |
| --- | --- | --- | --- | --- | --- | --- | --- | --- |
| CK | 48510548 | 7325092748 | 48207902 | 7186421139 | 0.0245 | 98.21 | 94.64 | 45.1 |
|  | 42584498 | 6430259198 | 42346140 | 6302930445 | 0.0243 | 98.31 | 94.91 | 45.17 |
|  | 42359110 | 6396225610 | 42074158 | 6265291659 | 0.0245 | 98.21 | 94.66 | 45.32 |
| LDH10 | 41101054 | 6206259154 | 40786728 | 6096215246 | 0.0251 | 97.97 | 94.04 | 44.67 |
|  | 44166496 | 6669140896 | 43775898 | 6545938763 | 0.0246 | 98.2 | 94.59 | 44.79 |
|  | 43584208 | 6581215408 | 43298236 | 6467574714 | 0.0244 | 98.27 | 94.8 | 44.96 |
| RW10 | 45502898 | 6870937598 | 45213816 | 6741222840 | 0.0247 | 98.16 | 94.53 | 44.34 |
|  | 43937072 | 6634497872 | 43516680 | 6483229044 | 0.0245 | 98.23 | 94.75 | 44.15 |
|  | 51579078 | 7788440778 | 51239140 | 7652400554 | 0.0245 | 98.23 | 94.7 | 44.41 |

Data filtering and quality assessment. RNA sequencing raw reads were filtered by the FASTP software. Clean reads were generated after removing low-quality reads, adaptor sequences, poly A and known non-coding RNAs from raw data.

Sample: Sample name. Raw reads: The total number of entries in the original sequencing data. Raw bases: The total amount of raw sequencing data. Clean reads: The total number of sequencing data entries after quality control. Clean bases: The total amount of sequencing data after quality control. Error rate (%): The average error rate of sequencing bases corresponding to quality control data, generally below 0.1%. Q20 (%) and Q30 (%): Quality evaluation is conducted on sequencing data after quality control. Q20 and Q30 refer to the percentage of bases with sequencing quality above 99% and 99.9%, respectively, in total bases. Generally, Q20 is above 85% and Q30 is above 80%. GC content (%): The percentage of the total G and C bases corresponding to the quality control data in the total base.
